# NN1213 – A Potent, Long-Acting, and Selective Analog of Human Amylin

**DOI:** 10.1021/acs.jmedchem.4c00022

**Published:** 2024-07-03

**Authors:** Kirsten Dahl, Kirsten Raun, Jakob Lerche Hansen, Christian Poulsen, Charlotta D. de la Cour, Trine Ryberg Clausen, Ann Maria Kruse Hansen, Linu M. John, Annette Plesner, Gao Sun, Morten Schlein, Rikke Bjerring Skyggebjerg, Thomas Kruse

**Affiliations:** † Novo Nordisk A/S, Novo Nordisk Park, DK-2760 Maaloev, Denmark; ‡ Novo Nordisk China, Novo Nordisk Research Center China, Building 2, 20 Life Science Park Road, Changping District, 102206 Beijing, China

## Abstract

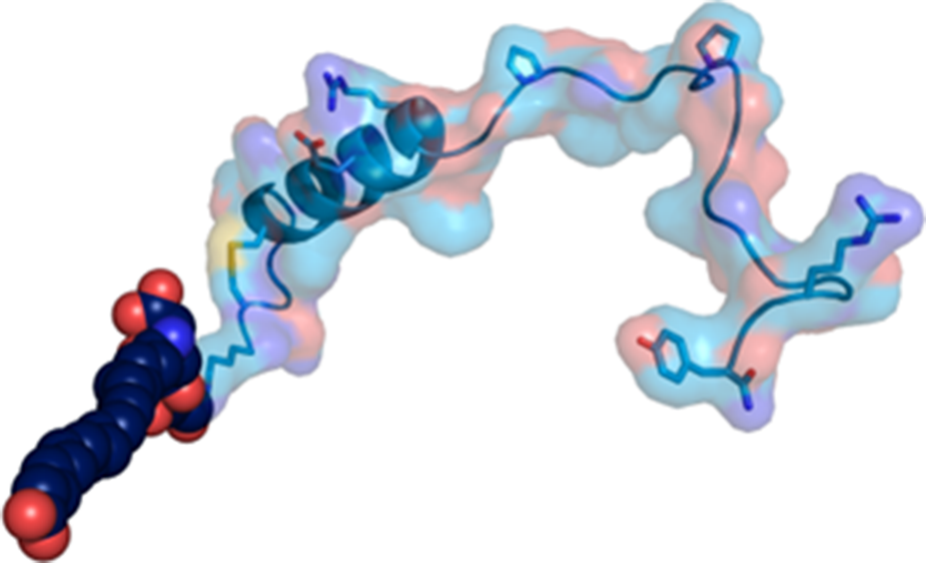

Amylin, a member of the calcitonin family, acts via amylin receptors in the hindbrain and hypothalamus to suppress appetite. Native ligands of these receptors are peptides with short half-lives. Conjugating fatty acids to these peptides can increase their half-lives. The long-acting human amylin analog, NN1213, was generated from structure–activity efforts optimizing solubility, stability, receptor affinity, and selectivity, as well as in vivo potency and clearance. In both rats and dogs, a single dose of NN1213 reduced appetite in a dose-dependent manner and with a long duration of action. Consistent with the effect on appetite, studies in obese rats demonstrated that daily NN1213 dosing resulted in a dose-dependent reduction in body weight over a 21-day period. Magnetic resonance imaging indicated that this was primarily driven by loss of fat mass. Based on these data, NN1213 could be considered an attractive option for weight management in the clinical setting.

## Introduction

Amylin is a 37 amino acid pancreatic peptide costored and cosecreted with insulin in response to nutrient intake.^1,2^ Upon secretion, amylin activates receptors in the hindbrain and hypothalamus to induce satiation, and it also impacts glucose control by postprandial suppression of nutrient-mediated glucagon secretion (not in the state of hypoglycemia) and by delayed gastric emptying.^2−5^ These biological effects support the investigation and development of optimized amylin analogs for use as therapeutics for weight management and diabetes. Indeed, the amylin analog pramlintide (Symlin) has been commercially available in the US market since 2005, as an add-on to insulin for people with diabetes who need additional support for glucose control.^6^ Additionally, the long-acting but nonselective amylin analog cagrilintide^7^ has been shown to induce significant weight loss, both alone^8^ and in combination with the long-acting glucagon-like peptide (GLP-1) analog semaglutide.^9,10^

Amylin belongs to the calcitonin family of peptides, which also includes calcitonin, α and β calcitonin gene-related peptide (CGRP), adrenomedullin 1 (AM1), and adrenomedullin 2 (AM2), also known as intermedin. These peptides are ligands to a complex family of receptors consisting of either the calcitonin receptor (CTR) or the calcitonin receptor-like receptor (CRLR) combined with one of three receptor activity-modifying proteins (RAMP1–3).^11^ The CRLR alone is not a functional receptor, but together with RAMP1, 2, or 3 it forms the CGRP receptor (CGRPR), AM1 receptor (AM_1_R), and AM2 receptor (AM_2_R), respectively. In contrast to CRLR, the CTR is a functional receptor on its own, where it preferentially responds to calcitonin.^11−13^ While native amylin can bind to the CTR, its affinity is greatly increased when the CTR is complexed with RAMP1 (amylin-1 receptor [AMY_1_R]), RAMP2 (amylin-2 receptor [AMY_2_R]), or RAMP3 (amylin-3 receptor [AMY_3_R]). Many of the peptides from the calcitonin peptide family can activate all of the CTR ± RAMP combinations, albeit with different potencies.^1,4,14,15^ The calcitonin family of peptides and receptors have been associated with numerous biological actions, including regulation of blood flow, bone metabolism, and energy metabolism.^4^

Salmon calcitonin (sCT) has ∼50% sequence similarity to mammalian calcitonin and is chemically more stable with a low tendency to form fibrils. sCT is a potent and nonselective agonist for both AMYRs and CTRs. It is marketed as Miacalcin (US)/Miacalcic (EU) and has been used for decades to treat postmenopausal osteoporosis, Paget’s disease, and other diseases of bone. As it was hypothesized that the beneficial effects regarding energy homeostasis were mediated via the amylin receptors (AMYRs), the analogs in the present program were counter-screened for potency at the related receptors, especially with regard to the CTR in order to generate AMYR-selective human amylin analogs. However, it is currently not known with certainty if and to what extent CTR agonism adds to the beneficial effects of an amylin analog.

Apart from AMYR selectivity, the inherent instability of the amylin peptide, including propensity toward fibril formation and chemical degradation, also needed to be addressed to achieve a molecule with drug-like characteristics.

The amino acid sequence of human amylin enables a process of misfolding whereby monomeric amylin initially forms soluble beta-sheet-rich oligomers that may be cytotoxic. Over time, these oligomers may mature further into elongated structures with a high content of beta-sheet strands and finally generate insoluble protein aggregates that are histologically visible as amyloid fibrils in islets of patients with type 2 diabetes.^1^ Some of these toxic oligomeric species are associated with beta-cell death and the progression of type 2 diabetes,^16−18^ and prevention of fibril formation has been associated with improved beta-cell survival and glucose control in preclinical models.^19,20^ Amylins from certain other mammals have a reduced tendency to form fibrils–one example being rat amylin, which differs from human amylin by six amino acids and includes the presence of three prolines.^21^ This feature from rat amylin has been utilized during the development of the commercially available amylin analog pramlintide, which has prolines in positions 25, 28, and 29, while the rest of the peptide is identical to human amylin (Table 1). The prolines thus greatly enhance the physical stability of pramlintide.

**Table 1:** Amino Acid Sequences of Established Ligands for the CTR^a^

peptide/aa#	1–10	11–20	21–30	31−
human amylin	KCNTATCATQ	RLANFLVHSS	NNFGAILSST	NVGSNTY-amide
rat amylin	KCNTATCATQ	RLANFLV**R**SS	NN**L**G**PV**L**PP**T	NVGSNTY-amide
pramlintide	KCNTATCATQ	RLANFLVHSS	NNFG**P**IL**PP**T	NVGSNTY-amide
sCT	CSNLSTCVLG	KLSQELHKLQ	TYPRTNTGSG	TP-amide

a **The bold** font represents a mutation relative to human amylin while the underlined font indicates a chemically modified residue. The amino acid sequence for cagrilintide has been published previously: 1–10: KCNTATCATQ; 11–20: RLA**E**FL**R**HSS; 21–30: NNFG**P**IL**PP**T; 31−: NVGSNT**P**-amide.^7^

The major chemical degradation products of pramlintide have been identified to include deamidation and isomerization of asparagine residues in positions 3, 21, and 22 and to a lesser degree in positions 14 and 35.^22^ Reduction of these deamidation reactions in pramlintide has been obtained by acidic formulation.^23^ Pramlintide has a short half-life in plasma of around 30–50 min^24^ and, consequently, must be injected prior to meals three times a day to be effective. Hence, in addition to selectivity and physical and chemical stability, a longer plasma half-life could be desirable for an AMYR agonist drug.

Many of the members of the calcitonin peptide family in mammals have a short half-life (in min) due to enzymatic degradation and kidney excretion. Conjugation of fatty acids to peptides has been demonstrated to convey albumin binding that, in the best cases, can protect the peptide from renal clearance and increase the plasma half-life from min to weeks, thereby hugely improving the convenience of a drug candidate. Semaglutide^25^ is one such example in the GLP-1 field, and a similar improvement could be anticipated for amylin using this fatty acid conjugation method. However, the addition of an albumin-binding moiety may, in many cases, reduce the receptor affinity and alter selectivity. In addition, it may affect the propensity to fibrillate. Therefore, the optimal positioning of this moiety on the peptide backbone must be thoroughly evaluated. With the recent discovery of cagrilintide, the clearance from plasma has been reduced with lipidation of the lysine in position 1.^7^ Cagrilintide is an agonist that binds to both the AMYR and the CTR.^7^ Finally, to minimize the risk of excessive immunogenicity, differences in amino acid sequence relative to human amylin can be limited. In summary, in the pursuit of a selective amylin analog, the key features to be incorporated in the molecule are a high ratio between the half maximal effective concentration (EC_50_) for AMY_3_R to CTR; chemical stability; low tendency to form fibrils; high solubility; and a long physiologic half-life to allow relevant exposure, while maintaining as much similarity to the human amylin molecule as possible.

Here, we report the generation of a selective amylin analog from a stepwise screening program addressing the above-mentioned challenges, with a description of its in vitro and in vivo characteristics. A screening plan, as described in the Experimental Section, was constructed to identify the optimal candidate among the above-mentioned parameters.

## Results

### SAR and Structure

Two series of peptides were generated and characterized for solubility, tendency to form fibrils, and in vitro characteristics. A subset of the synthesized peptides from the second series was further characterized with regard to in vivo efficacy (reduction of food intake). The inclusion of proline in position 21 confers several benefits: improvement of physical stability, since proline appears to be less likely to contribute to beta-sheet structures; replacement of chemically unstable asparagine in this position; and compatibility with selectivity.

The first series of nine peptides were synthesized based on the human amylin scaffold, aiming at formulation at neutral pH (Table 2). The amylin scaffold appeared to be the best starting point for achieving selectivity toward the AMYR since pramlintide already possesses some selectivity. All compounds had a disulfide bridge from Cys2–Cys7, similar to human amylin, and were acylated with an albumin binder on the side chain amine of Lys1. Peptides **1** and **2** were acylated with C20diacid-gGlu- and **3**–**9** with C20diacid-gGlu-gGlu-.

**Table 2:** Structures of the Peptides 1–9 Aiming for Neutral Formulation

peptide	1–10	11–20	21–30	31−
h-amylin	KCNTATCATQ	RLANFLVHSS	NNFGAILSST	NVGSNTY-amide
1	KCNTATCATQ	RLADFLRHSS	QNFGAPLSST	PVGSDTY-amide
2	KCNTATCATQ	RLADFLRHSS	QNFGAPLSST	NVGSPTY-amide
3	KCNTATCATQ	RLADFLRHSS	PNFGPILSST	NVGSDTY-amide
4	KCNTATCATQ	RLADFLRHSS	NPFGPILSST	NVGSDTY-amide
5	KCNTATCATQ	RLADFLRHSS	PNFGAPLSST	NVGSDTY-amide
6	KCNTATCATQ	RLADFLRHSS	PNFGAIPSST	NVGSDTY-amide
7	KCNTATCATQ	RLADFLRHSS	NPFGAPLSST	NVGSDTY-amide
8	KCNTATCATQ	RLADFLRHSS	NPFGAIPSST	NVGSDTY-amide
9	KCNTATCATQ	RLADFLRHSS	ENFGPILSST	PVGSDTY-amide

Peptides **1**–**9** were screened in vitro, and receptor potency toward the human AMY_3_R was evaluated using a luciferase assay (Table 3). Selectivity toward the human AMYR was evaluated by the ratio between IC_50_ on the human AMY_3_R versus the human CTR (see Table 4). The rat AMYR was included in the in vitro evaluation to guide the interpretation of results from the rat screening model.

**Table 3:** In Vitro hCTR and hAMY_3_R Functional Results and Selectivity Ratios for Analogs 1–9^a^

	functional assays					
	luciferase (human receptor assay)			cAMP (rat receptor assay)		
	hAMY_3_R	hCTR	ratio	rAMY_3_R	rCTR	ratio
	EC_50_ (pM)	EC_50_ (pM)	hCTR/hAMY_3_R	EC_50_ (pM)	EC_50_ (pM)	rCTR/rAMY_3_R
pramlintide	5	70	14	4	1822	455
sCT	1.6	2.9	1.8	0.52	0.14	0.27
1	180	697	3.9	463	14,340	31
2	1535	1371	0.9			
3	319	3005	9.4			
4	267	672	2.5			
5	219	2579	12			
6	161	2477	15	643	860,600	1338
7	200	1380	6.9			
8	390	2190	5.6			
9	1061	8309	7.8			

a Data are EC_50_ for AMY_3_R and CTR and corresponding selectivity ratios. Additional results including EC_50_ and negative logarithm of the EC_50_ (pEC_50_) values, 95% confidence intervals (CIs), and number of experiments are reported in Supporting Information (Table S12). Data for cagrilintide have been published previously^7^ and are shown here for reference: luciferase (human receptor assay) EC_50_ values (pM) were hAMY_3_R 49, hCTR 62, ratio 1.3; cAMP (rat receptor assay) EC_50_ values (pM) were rAMY_3_R 348, rCTR 287, ratio 0.8. hAMY_3_R, human AMY_3_R; hCTR, human CTR; rAMY_3_R, rat AMY_3_R; rCTR, rat CTR.

**Table 4:** In Vitro hCTR and hAMY_3_R Binding Results and Selectivity Ratios for Analogs 3, 5–7^a^

	binding assays					
analog	human receptors			rat receptors		
	hAMY_3_R	hCTR	ratio	rAMY_3_R	rCTR	ratio
	IC_50_ (pM)	IC_50_ (pM)	hCTR/hAMY_3_R	IC_50_ (pM)	IC_50_ (pM)	rCTR/rAMY_3_R
pramlintide	114	1492	13	122	5834	48
sCT	89	66	0.7	28	30	1.1
3	5095	74,108	14	4690	355,750	76
5	1121	30,233	27	1300	105,572	81
6	355	19,476	55	217	96,915	447
7	1125	11,662	10	1002	7545	7.5

a Data are IC_50_ for AMY_3_R and CTR and corresponding selectivity ratios. Additional results including IC_50_ and negative logarithm of the half maximal inhibitory concentration of the peptides (pIC_50_) values, 95% CIs, and number of experiments are reported in Supporting Information (Tables S12 and S13). Data for cagrilintide have been published previously^7^ and are shown here for reference: binding assay (human receptors) IC_50_ values (pM) were hAMY_3_R 170, hCTR 233, ratio 1.3; binding assay (rat receptors) IC_50_ values (pM) were rAMY_3_R 520, rCTR 681, ratio 1.3.

Furthermore, peptides **1**–**7** were evaluated for their solubility at pH 3 through pH 8 (Table 5) and stability against fibril formation in the thioflavin T (ThT) assay (Table 6 and Supporting Information). In particular, peptide **6** seemed to combine acceptable potency with some selectivity, and it was decided to further pursue the proline residues at positions 21 and 27 as they exhibited the best selectivity. In general, it was observed that the C-terminal tyrosine amide was essential for selectivity. The proline in position 21 was also attractive since it substitutes the chemically unstable asparagine while at the same time conveying physical stability into the molecule. Not all peptides possessed an adequate solubility profile, and it was found that the chemical stability at neutral pH required for a drug candidate was not sufficient among peptides **1**–**7**. This was partly related to deamidation and isomerization at asparagine residues but also the disulfide bridge. Degradation of the asparagine residues and general stability were significantly improved at pH 4; however, formulation at pH 4 would be close to the isoelectric point (pI) of some of the compounds, potentially leading to precipitation issues. Of particular risk was precipitation at higher concentrations, or potential issues with irreversible precipitation at the injection site when the pI was passed, as has been observed with analogs previously reported.^7^ Consequently, the focus was directed to ensure a pI in the neutral to the basic range, and a new series of compounds were designed with higher pI but with the intention for formulation at pH 4 (Table 7).

**Table 5:** Solubility Profile of Analogs 1–7^a^

analog	pH 3	pH 4	pH 5	pH 6	pH 6.5	pH 7	pH 7.5	pH 8	calculated pI
1	200	200	200	200	200	200	200	200	5.8
2	200	200	200	200	75	7	7	15	7.1
3	200	1	1	1	1	1	1	2	4.8
4	200	200	38	173	200	200	200	200	4.8
5	200	200	172	200	200	200	200	200	4.8
6	200	200	200	200	190	200	200	200	4.8
7	200	96	141	189	200	195	200	200	4.8

a Solubility values are stated as μM and “200” means fully soluble at 200 μM.

**Table 6:** Fibril Formation Propensity, Peptide Recovery (Filtration Followed by HPLC) After 45 h of Shaking, and HMWP Content of Analogs 1–9 (ThT Assay)^a^

analog	ThT, pH 7.5 lag-time (h)	recovery (%)	HMWP (%)
1	>45	100	7
2	0	0	ND
3	0.3	19	24
4	4	21	5
5	35	100	7
6	>45	100	2.5
7	>45	100	3
8	>45	100	2.5
9	>45	100	6

a HMWP, high molecular weight product; ND, not done.

**Table 7:** Analogs 10–21 with Anticipated Acidic Formulation pH

	1–10	11–20	21–30	31−
h-amylin	KCNTATCATQ	RLANFLVHSS	NNFGAILSST	NVGSNTY-amide
10	KCNTATCATQ	RLANFLRHSS	PNFGAIPSST	NVGSNTY-amide
11	KCNTATCATQ	RLANFLRHSS	PNFGAIPSST	PVGSETY-amide
12	KCNTATCATQ	RLAEFLRHSS	PNFGAIPSST	NVGPNTY-amide
13	KCNTATCATQ	RLANFLRHSS	PNFGAIPSST	NVGSKTY-amide
14	KCNTATCATQ	RLANFLRHSS	PNFGAIPSST	NVGSKTY-amide
15	KCNTATCATQ	RLANFLRHSS	PNFGAIPSST	NVGSRTY-amide
16	KCNTATCATQ	RLANFLRHSS	PNFGAIPSST	NVGRNTY-amide
17	KCNTATCATQ	RLANFLRHSS	PNFGAIPSST	NVGHNTY-amide
18	KCNTATCATQ	RLANFLRHSS	PNFGAIPSST	NVGSHTY-amide
19	RCNTATCATQ	RLANFLRHSS	PNFGAIPSST	NVGSHTY-amide
20	KCNTATCATQ	RLAEFLRHSS	PNFGAIPSST	NVGSRTY-amide
21	KCNTATCATQ	RLADFLRHSS	PNFGAIPSST	NVGSRTY-amide

All peptides **11**–**21** had a disulfide bridge from Cys2–Cys7 and were acylated with an albumin binder on either side chain amine (epsilon) or N-terminus (alpha) of Lys1 (Arg1 for **19**). Peptide **10** was acylated with C20diacid-gGlu—OEG-OEG- on alpha; **11**, **12**, **13**, **16**, and **17** with C20diacid-gGlu- on alpha; **14** and **20** with C20diacid-gGlu- on epsilon; **15** and **18** with C20diacid-gGlu-OEG-; **19** with C20diacid-gGlu-Gly- on alpha; and **21** with C20diacid-gGlu-gGlu- on epsilon.

Peptides **10**–**21** were tested with regard to solubility (Table 8) and physical stability (Table 9), and it was apparent that by shifting the pI, promising screening characteristics were successfully achieved for the majority of the compounds.

**Table 8:** Solubility Profile of Analogs 10–21^a^

analog	pH 3	pH 4	pH 5	pH 6	pH 6.5	pH 7	pH 7.5	pH 8	calculated pI
10	200	200	200	200	200	200	200	200	10.1
11	200	200	200	200	200	200	200	200	8.2
12	200	200	200	200	200	200	200	200	8.2
13	200	200	200	200	200	200	200	200	10.6
14	200	200	200	200	200	200	200	200	10.2
15	200	200	200	200	200	200	200	200	11.4
16	200	200	200	200	200	200	200	200	11.7
17	200	200	200	200	200	200	200	200	10.1
18	200	200	200	200	200	200	200	200	8.8
19	200	200	200	200	200	200	50	68	11.4
20	200	200	200	200	200	200	183	109	8.8
21	200	200	200	200	200	155	106	110	7.2

a Solubility values are stated as μM and “200” means fully soluble at 200 μM.

**Table 9:** Fibril Formation Propensity, Recovery (Filtration Followed by HPLC) After 45 h of Shaking, and HMWP Content for Analogs 10–21 (ThT Assay)

analog	ThT, pH 4.0 lag-time (h)	recovery (%)	HMWP (%)
10	34	0	2.7
11	40	74	2.6
12	>45	78	3.9
13	35	99	2.1
14	>45	100	3.0
15	27	67	0.7
16	>45	100	2.2
17	26	92	1.7
18	15	30	3.5
19	>45	84	0.3
20	7	91	4.9
21	42	96	0.9

From the in vitro data (Tables 10 and 11), it was apparent that selectivity was achieved for several peptides (**10**, **14**, **15**, **16**, **17**, **18**, **20**, and **21**). From the ThT assay (Table 9 and Supporting Information), only three of the AMYR selective peptides (**14**, **16**, and **21**) were able to resist physical stress for more than 40 h. Two peptides (**15** and **21**) had a selectivity ratio above 30. Peptide **6** and peptides **11**–**21** with neutral/high pI were chosen for further evaluation in vivo to evaluate their ability to reduce food intake in rats after a single administration and to have increased insights into the duration of effects from the peptides in vivo (Table 11).

**Table 10:** In Vitro Potency for Human and Rat AMY_3_R and CTR, and Selectivity Ratio; Analogs 10–21^a^

	functional assays					
analog	luciferase (human receptor assay)			cAMP (rat receptor assay)		
	hAMY_3_R	hCTR	ratio	rAMY_3_R	rCTR	ratio
	EC_50_ (pM)	EC_50_ (pM)	hCTR/hAMY_3_R	EC_50_ (pM)	EC_50_ (pM)	rCTR/rAMY_3_R
10	432	10,000	23	1788	141,900	79
11	186	1993	11	1288	16,210	13
12	47	314	6.7			
13	310	2609	8.4	599	30,370	50
14	364	5459	15	754	37,160	49
15	352	18,260	52			
16	217	2522	12			
17	151	2492	17	926	16,810	18
18	350	6500	19			
19	222	1322	6			
20	340	5204	15			
21	177	5210	29	262	102,500	391

a Data are EC_50_ for AMY_3_R and CTR and corresponding selectivity ratios. Additional results including EC_50_ and pEC_50_ values, 95% CIs, and number of experiments are reported in Supporting Information (Table S14).

**Table 11:** In Vitro Binding Affinity for Human and Rat AMY_3_R and CTR and Selectivity Ratio; Analogs 10–21^a^

	binding assays					
	human receptors			rat receptors		
	hAMY_3_R	hCTR	ratio	rAMY_3_R	rCTR	ratio
analog	IC_50_ (pM)	IC_50_ (pM)	hCTR/hAMY_3_R	IC_50_ (pM)	IC_50_ (pM)	rCTR/rAMY_3_R
10	1138	102,400	90	3006	349,700	116
11	589	11,927	20	1171	51,410	44
12	100	1088	11	176	5976	34
13	1139	73,390	64	1715	89,170	52
14	885	42,342	48	920	55,644	60
15	213	21,148	99	372	32,737	88
16	662	19,280	29	1768	96,440	55
17	484	14,097	29	1293	49,815	39
18	2746	52,262	19	1240	71,016	57
19	344	15,460	45	203	17,110	84
20	745	22,905	31	449	50,048	111
21	561	42,049	74	290	134,993	465

a Data are IC_50_ for AMY_3_R and CTR and corresponding selectivity ratios. Additional results including IC_50_ and pIC_50_ values, 95% CIs, and number of experiments are reported in Supporting Information (Table S15).

Data are mean ± SEM measured as percent food intake relative to vehicle in rats after a single sc injection of 30 nmol/kg of the test substance. Data for cagrilintide have been published previously^7^ giving rise to 85% and 84% reduction of food intake in the periods from 0−24 h and 24–48 h, respectively. SEM is the standard error of the mean.

**Figure 1:**
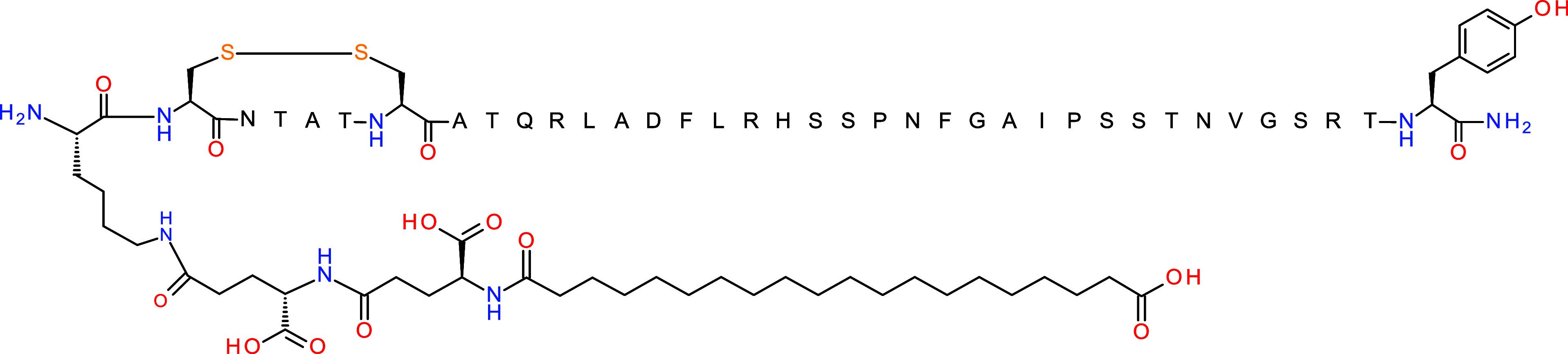
Amino acid sequence of a selective amylin analog, NN1213 (**21)**, with acylation.

The in vivo screening that tested the ability of the peptides to reduce appetite over periods of up to 48 h in rats after a single sc injection showed that although peptides **6**, **11**, **12**, **14**, **15**, **17**, **18**, **19**, **20**, and **21** were able to reduce appetite by approximately 50% or more after the first 24 h, only peptides **6**, **11**, **17**, **20**, and **21** were able to have a sustained effect from 24 to 48 h after a single injection (Table 12). There was a trend toward a shorter duration of action when the pI was higher, possibly indicating reduced albumin binding for positively charged compounds; however, from the screening program, it appeared that peptide **21** was especially attractive due to being both selective and exhibiting a long duration of effect. Peptide **21** thus represented an optimum within this series with regard to combining physical and chemical stability with AMYR selectivity and promising in vivo efficacy. Hence, peptide **21** (Figure 1) was chosen for further in vitro and in vivo characterization and is hereafter referred to as **NN1213**.

**Table 12:** Pharmacodynamic Screening: Acute Food Intake Reduction in Rats

analog	0–24 h (%)	24–48 h (%)
6	72 ± 2	62 ± 3
10	22 ± 5	1 ± 5
11	49 ± 2	38 ± 2
12	62 ± 6	7 ± 7
13	37 ± 7	25 ± 7
14	51 ± 2	15 ± 4
15	47 ± 4	0 ± 5
16	32 ± 4	4 ± 5
17	51 ± 8	28 ± 3
18	51 ± 3	3 ± 6
19	59 ± 4	6 ± 7
20	52 ± 6	30 ± 7
21	70 ± 5	50 ± 1

### Characterization of NN1213 and Comparison to Cagrilintide, Pramlintide, and sCT

#### In Vitro Evaluation

NN1213 was assessed to be more potent on the human AMY_3_R compared to the human CTR in the luciferase screening assays.

To further characterize NN1213, the potency of NN1213 on other receptors in the calcitonin family was investigated. Henrietta Lacks (HeLa) cells were transduced with the full range of human calcitonin family receptors (see Supporting Information S1: BacMam Functional Assay Methods (Human) for further information) and used to determine the potency of NN1213 in functional in vitro receptor assays by measuring the accumulation of cAMP.

Cagrilintide, pramlintide, and sCT were included as reference compounds for comparison, and native endogenous ligands were included as pharmacological tools to verify receptor functionality following the coexpression of CTR or CRLR with RAMPs (Table 4).

**Figure 2:**
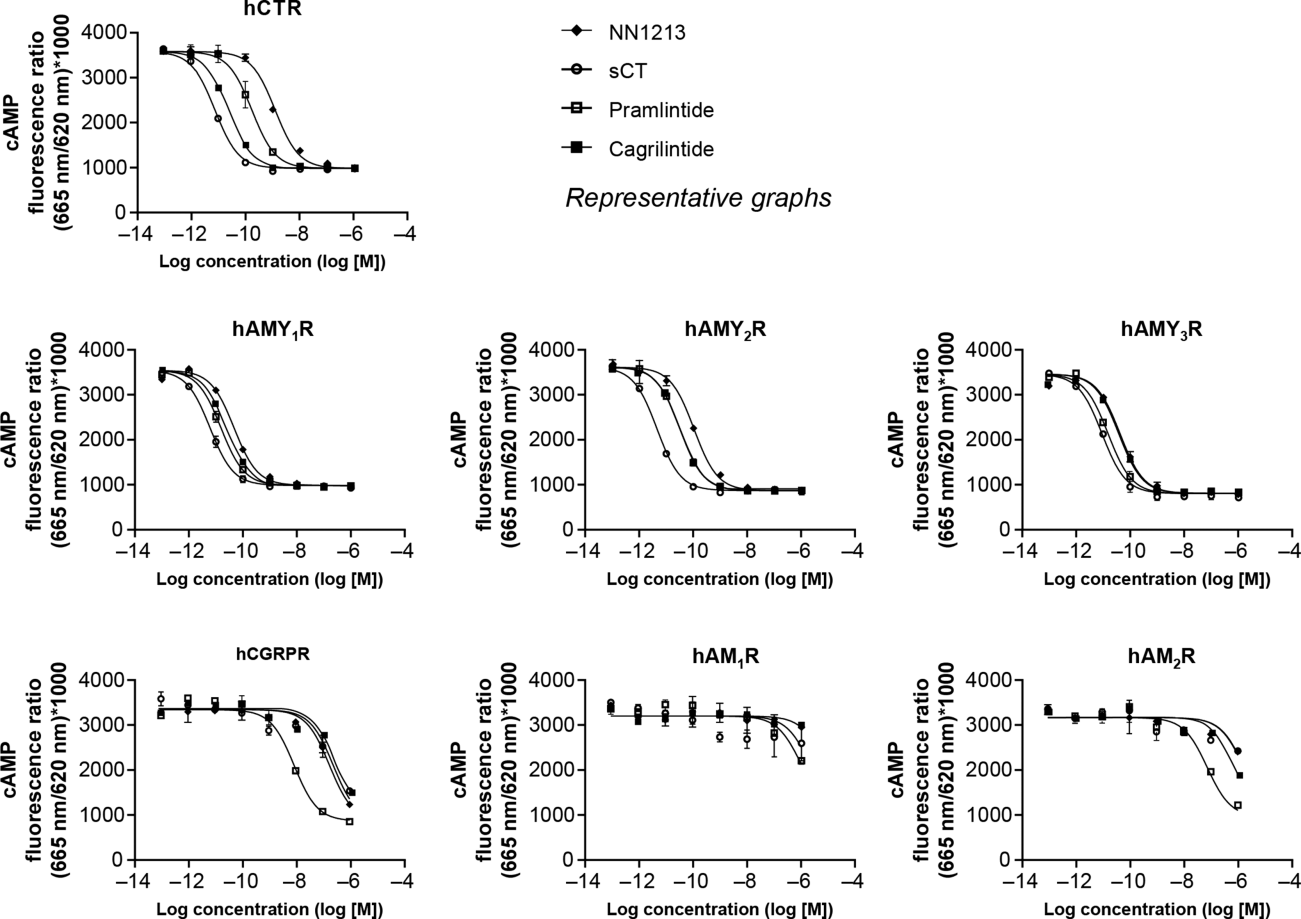
In vitro potency of NN1213, cagrilintide, pramlintide, and sCT on human calcitonin family receptors. Error bars are 95% CIs. hAM_1_R, human AM_1_R; hAM_2_R, human AM_2_R; hAMY_1_R, human AMY_1_R; hAMY_2_R, human AMY_2_R; hCGRPR, human CGRPR; CI, confidence interval.

In these studies, NN1213 appeared to be an AMYR selective agonist with greater in vitro potency on human AMYRs compared to the human CTR (Table 13, Figure 2, Supporting Information S2: BacMam Functional Assay Results (Human), and Table S4). As expected, sCT was found to be a nonselective agonist that was highly potent on both CTR and AMYRs and was more potent on the CTR than both NN1213 and pramlintide. Additionally, NN1213, cagrilintide, and sCT displayed no or very low activity on human CGRP- and adrenomedullin receptors (AMR), whereas pramlintide seemed to have slightly more activity on CGRPR and AM_2_R when tested in high concentrations.

**Table 13:** In Vitro Potency of NN1213, Cagrilintide, Pramlintide, and sCT on Human Receptors

	NN1213	cagrilintide	pramlintide	salmon calcitonin
	pEC_50_ (95% CI)	pEC_50_ (95% CI)	pEC_50_ (95% CI)	pEC_50_ (95% CI)
CTR	8.76 (7.87–9.65)	10.64 (10.10–11.18)	9.48 (8.16–10.80)	11.12 (11.01–11.23)
AMY_1_R	10.24 (9.55–10.93)	10.73 (9.97–11.50)	10.66 (9.53–11.80)	11.21 (10.78–11.65)
AMY_2_R	9.74 (8.64–10.84)	10.37 (9.77–10.97)	10.19 (8.65–11.74)	11.26 (10.83–11.69)
AMY_3_R	10.12 (9.23–11.00)	10.33 (9.92–10.74)	10.53 (9.18–11.89)	10.87 (10.33–11.40)
CGRPR	6.57 (6.24–6.90)	6.25 (5.87^a^–6.63)	7.63 (6.78–8.49)	6.41 (5.90^a^–6.92)
AM_1_R	NA	NA	NA	NA
AM_2_R	NA	6.12 (5.85^a^–6.39)	6.83 (6.15–7.52)	NA

a Data below six are extrapolated out of range for test concentrations. *n* = 3 for CTR, AMYR; *n* = 4 for CGRPR, AMRs. NA, no activity.

Species-specific in vitro assays were also performed. Methods and results are provided in Supporting Information S3: Species-Specific In vitro Assay Methods and S4: Species-Specific In vitro Assay Results*.*

#### In Vivo Evaluation

The pharmacokinetic (PK) and pharmacodynamic efficacies of NN1213 were next investigated in diverse animal models to evaluate the potential for this analog as a weight management therapeutic.

#### Pharmacokinetic and Pharmacodynamics

**Figure 3:**
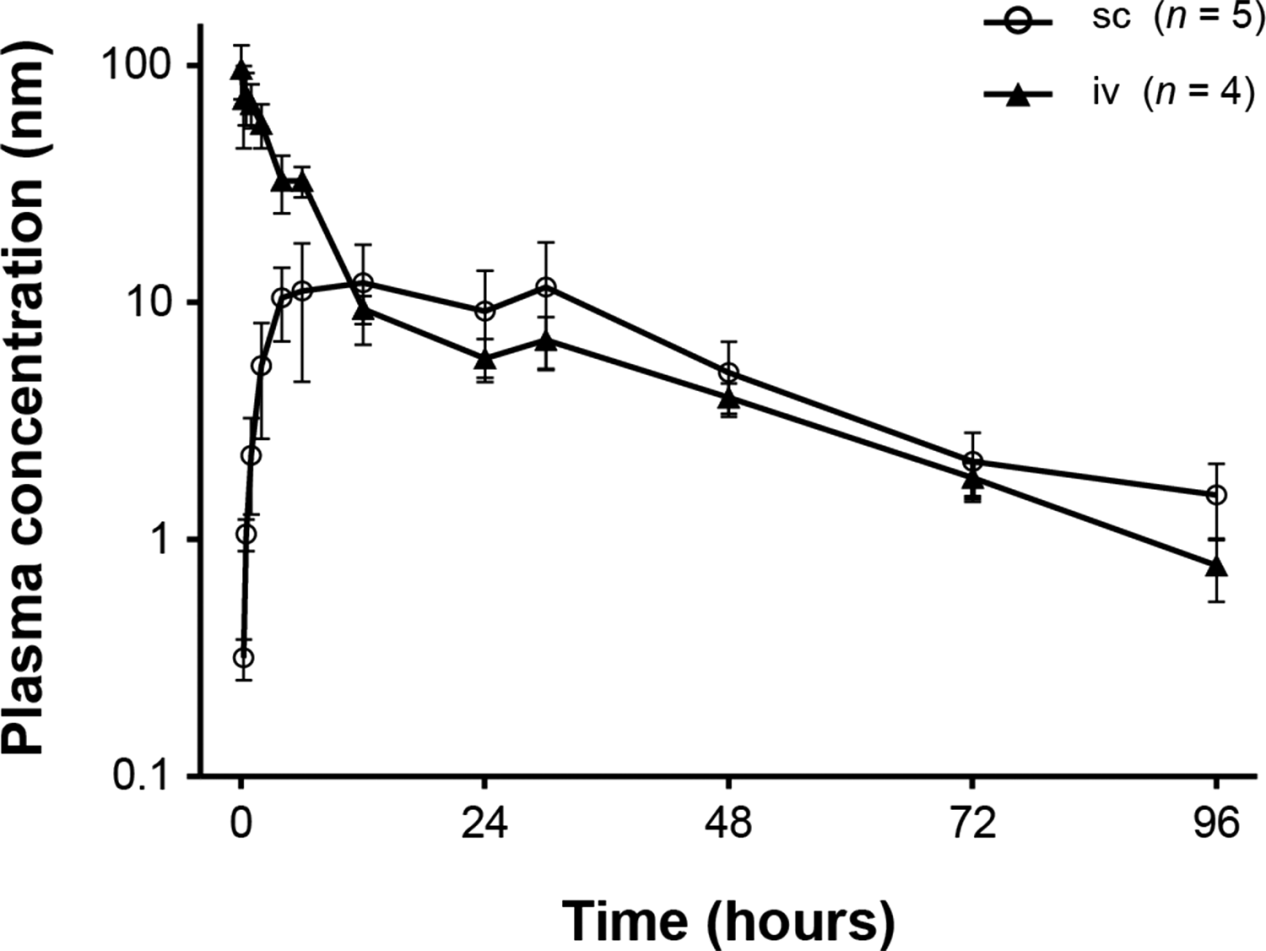
Mean plasma concentrations of NN1213 in rats following a single 2.5 nmol/kg dose. Error bars are ±SD. SD, standard deviation.

The PK properties of NN1213 were examined in rats, rabbits, dogs, and minipigs. In rats, the plasma terminal half-life was estimated to be approximately 24 h and the bioavailability after sc administration was approximately 40% (Figure 3).

The terminal plasma half-life was estimated to be approximately 50 h after iv administration of 2 nmol/kg in rabbits (*n* = 9), approximately 115 h after iv administration of 4.8 nmol/kg in Göttingen minipigs (*n* = 4), and approximately 76 h after iv administration of 6 nmol/kg in beagle dogs (*n* = 3) (Supporting Information, Table S9). Bioavailability was not determined in rabbits, minipigs, or dogs.

#### Acute Efficacy

**Figure 4:**
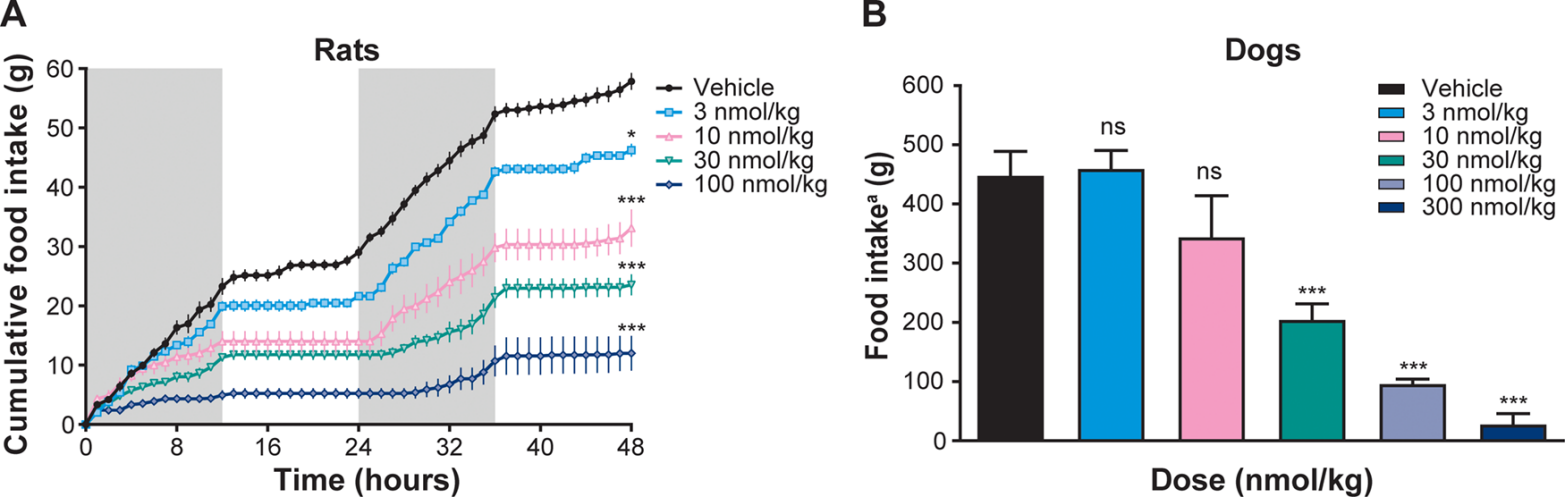
Acute food intake reduction in rats and dogs after a single sc dose of NN1213 in (A) rats and (B) dogs. The gray background in the acute rat food intake graph indicates a dark phase during which the rats are actively feeding. Error bars are ± SEM * = *p* < 0.05, *** = *p* < 0.001, ns = not significant. ^*a*^For dogs, food intake (four meals) was measured over 48 h after a single sc dose.

The ability of NN1213 to reduce appetite after a single administration was evaluated at 3–100 nmol/kg in rats and at 3–300 nmol/kg in dogs in comparison to vehicle-treated controls. In rats, a dose-dependent reduction in food intake with a long duration of action was apparent at all dose levels (Figure 4, left panel). At 3 nmol/kg, food intake was reduced by 25% compared to controls after the first 24 h and by 15% in the following 24 h. The high dose (100 nmol/kg) resulted in a sustained reduction of food intake of 70% both in the first 24 h as well as in the following 24 h, indicating a slow elimination from the body for NN1213. In dogs, NN1213 was also able to reduce appetite in a dose-dependent manner (Figure 4, right panel). The lowest dose (3 nmol/kg) did not affect appetite, whereas the highest dose (300 nmol/kg) reduced food intake by 90% for the first 48 h after a single sc administration. The effect was long-lasting: at the 30 nmol/kg dose, appetite suppression was present for at least 7 days. The administration of NN1213 at a dose ranging from 3 to 300 nmol/kg was not associated with any signs of discomfort in the dogs and no vomiting.

#### Subchronic Efficacy

**Figure 5:**
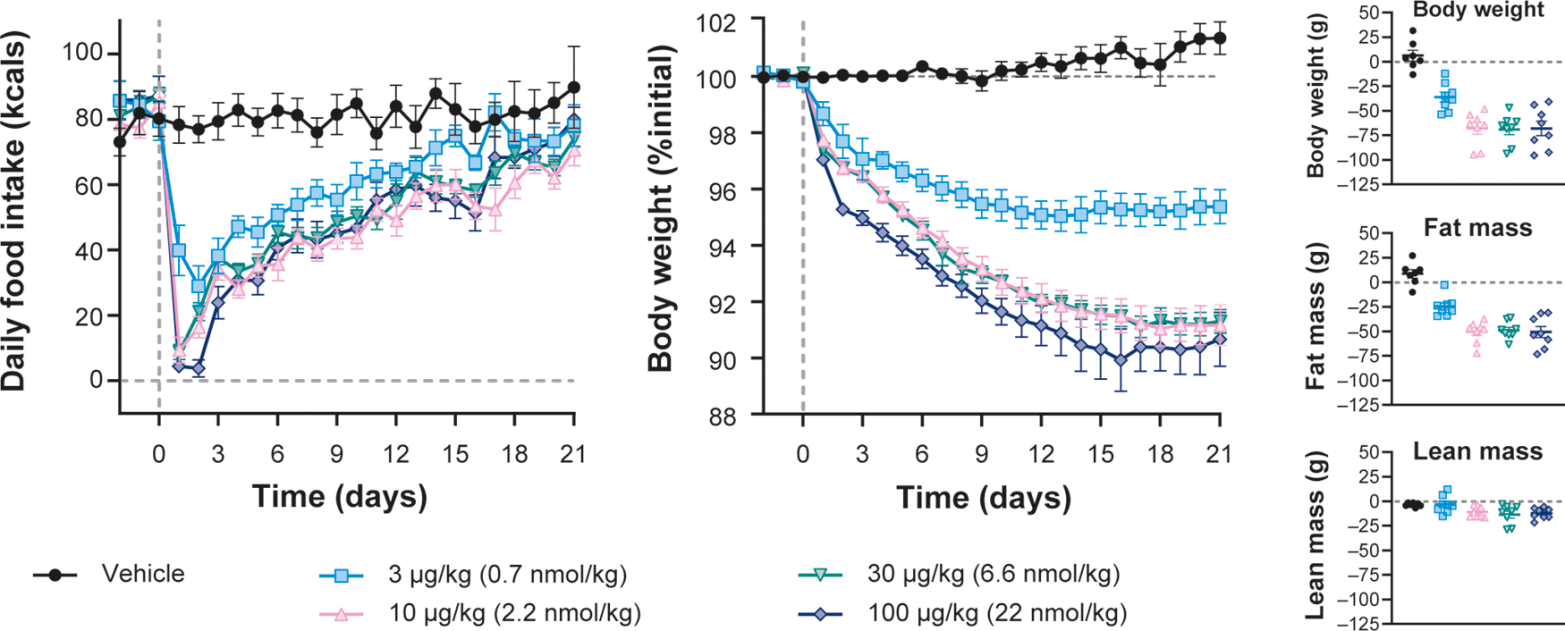
Food intake, body weight, and change in body composition in DIO rats dosed sc once daily with NN1213. Error bars are ± SEM. SEM, standard error of the mean.

The ability of NN1213 to reduce body weight with repeated dosing was tested in DIO rats fed a high-energy diet. DIO rats were sc injected once daily with NN1213 at dose levels of 0.7, 2.2, 6.6, and 22 nmol/kg (3, 10, 30, and 100 μg/kg, *n* = 8/group). Further details are provided in Supporting Information S5: In vivo Methods (DIO Rat Weight Loss Assay). Subchronic treatment of DIO rats with NN1213 transiently reduced food intake, with a maximal effect occurring during days 1–2, after which the suppressive effect slowly diminished with time (Figure 5, left panel). The reduction in food intake resulted in a 4.7% (at the 0.7 nmol/kg dose) and 8.7–9.3% (at the 2.2, 6.6, 22 nmol/kg doses) reduction in body weight relative to baseline (Figure 5, middle panel; Supporting Information, Table S10). The maximal reduction in daily food intake was observed around day 2. By approximately day 15, all NN1213 treatment groups had plateaued in body weight loss as food intake levels were closer to (although still slightly below) that of the DIO vehicle control group.

NN1213 reduced body weight in a dose-dependent manner, with the 2.2 and 22 nmol/kg dose levels resulting in absolute body weight levels on par with the body weight of the lean control group by the end of the treatment period. After 21 days, the rats in the low-dose group (0.7 nmol/kg) had lost 32.0 ± 3.6 g. In the same period, the high-dose group had lost 69.6 ± 7.5 g. The latter was a weight loss corresponding to 90.7% of their body weight prior to the first dose (Supporting Information, Table S10).

Body composition data were collected by magnetic resonance scans 6 days prior to the first dose and on day 21. Body weight, fat mass, and lean mass are shown as the change from baseline (Figure 5, right panels). Treatment with NN1213 induced a dose-dependent reduction in fat mass (Figure 5, right middle panel) that reflected the dose-dependent reduction in body weight (Figure 5, right top panel), with minimal effects on lean mass (Figure 5, right bottom panel), indicating that NN1213-mediated body weight loss is primarily driven by the loss of fat mass (Supporting Information, S5: In vivo Methods).

## Discussion and Conclusions

In this study, the design efforts, focused on identifying the minimal and optimal number of mutations to the human amylin scaffold to cause selectivity toward the AMYR versus the CTR, minimize fibril formation tendency at pH 4, ensure solubility in the pH range from 4 to 7.4, reduce chemical instability at pH 4, and provide good in vivo efficacy and long duration of action.

Having prolines in the sequence clearly offers protection from amyloid fibril formation. Indeed, proline is the only amino acid not contributing to the backbone-to-backbone amide interactions required for beta sheet-formation. Human amylin contains the sequence NFGAIL in position 22–27 (Table 14), which has been studied by Yan and colleagues in isolated form as particularly important for fibril formation.^26^

**Table 14:** Amino Acid Sequence of NN1213 Compared to Human and Rat Amylin

peptide	1	11	21	31
human amylin	KCNTATCATQ	RLANFLVHSS	NNFGAILSST	NVGSNTY-amide
rat amylin	KCNTATCATQ	RLANFLVRSS	NNLGPVLPPT	NVGSNTY-amide
NN1213	KCNTATCATQ	RLADFLRHSS	PNFGAIPSST	NVGSRTY-amide

The proline residue in position 25, but not 28 and 29, in rat amylin is within the sequence corresponding to the NFGAIL beta-strand in human amylin. Indeed, Abedini and Raleigh demonstrated that just one proline in position 26 is enough to protect against fibril formation.^27^ However, one proline may not be sufficient to protect against fibril formation if peptide aggregation is enhanced by adding a fatty acid to the peptide. In accordance with previous findings, where a C-terminal proline and salt bridge were introduced to the cagrilintide molecule to improve stability,^7^ results indicated that (i) prevention of fibril formation of the C20 diacid peptide needed more than one proline replacement; (ii) a second proline did not need to be in the NFGAIL region; and (iii) additional regions to be investigated were the noncharged, lipophilic LANFLV-region from 12 to 17 where His in position 17 reduced fibrillation. This led to the strategy of utilizing the two prolines to remedy the fibril formation issue, one in the NFGAIL region and the other in a position, where it could benefit chemical stability–notably the Asn21 Asn22 sequence, since a neutral formulation initially was preferred, and then combining with the addition of charge in the 12–17 region. Thus, protection against fibril formation was achieved by keeping the prolines in the peptide backbone but changing the positions compared to rat amylin. As the positioning of prolines may have a large impact on the tertiary structure, receptor potency and selectivity were thoroughly investigated and NN1213 was found to be a selective and potent agonist for AMYRs.

Initially, it seemed likely that a neutral formulation was possible and that an acidic pI (obtained by addition of negatively charged residues) would be of the most benefit. For this, the most unstable deamidation sites needed modification. However, as development of a neutral formulation not only caused deamidation but also involved disulfide bridge instability, it was decided to revert to using an acidic formulation. For this purpose, a basic or neutral pI was thought to be more suitable since the pH change from 4 to neutral at the injection site would not involve passing the pI. Stability data for peptides **1**–**9** with acidic pI also provided the valuable insight that the Pro21,27 combination with prolines on each side of the beta-strand NFGAIL region improved selectivity and fibril formation propensity without causing an unacceptable decrease in in vitro and in vivo potency. In addition, the proline in position 21 serves to reduce deamidation since it substitutes an asparagine that is particularly prone to deamidate.^22^ A salt bridge from positions 14–17 supported an alpha helix in this region and counteracted a tendency to fibril formation, and the aspartic acid in position 14 replacing the deamidation-prone asparagine was compatible with the desired in vitro characteristics. Finally, NN1213 had a long duration of action in the rat food intake model. Based on these results, NN1213 was chosen for further in vitro and in vivo characterization.

Characterization of NN1213 confirmed that it was possible to identify a peptide with attractive formulation characteristics and with selectivity toward the AMYR over the CTR. From in vivo characterization, NN1213 also proved to be effective in lowering appetite in rats and dogs, and lowering body weight in DIO dogs, supporting reductions in appetite and body weight observed in mice from previous published studies of NN1213.^28^ The current literature suggests that these effects are centrally mediated, whereby amylin activation of the central reward areas (ventral tegmental area [VTA] and nucleus accumbens) appears to have a reduced effect on food reward. Further evidence suggests that amylin and leptin may interact at different sites (caudal hindbrain, hypothalamus, and VTA), resulting in reduced food intake and increased energy expenditure, as indicated in the literature from both rodent and human studies.^29,30^

From the daily consumption of food observed in the DIO rats, the steep decrease around day 2 is a typical pattern observed with appetite-reducing agents in rodents.^31^ The reduction in food intake in rats was 25% with NN1213 at 3 nmol/kg, compared to a 45% reduction with the same dose of the amylin analog cagrilintide^7^ despite comparable plasma half-lives. This could indicate that with the selectivity toward the AMYR at the expense of the CTR, some efficacy was lost, which was also supported by the in vitro data; for example, cagrilintide was 3-fold more potent than NN1213 on the human AMY_3_R assessed in the in vitro binding assay.^7^ Beneficial effects from CTR agonism on body weight have also been suggested by the literature,^32−34^ but it remains to be fully elucidated how much of the effect pertains to the AMYR versus CTR.^35,36^ Similarly, it could be speculated if AMYR and CTR agonism contributes differently to nausea and whether the relative increased time for dissociation of the agonist from the receptor (off-rate) reported for some CTR agonists is the underlying reason.^37^ However, the translation across species, with regards to both beneficial and adverse effects, continues to offer challenges in the differentiation attributable to the individual receptors of the calcitonin family, in this context the CTR and the AMYR.^28,38^

In conclusion, the human amylin analog NN1213 reduced appetite in a dose-dependent manner and resulted in a dose-dependent body weight reduction in obese rats over 21 days. Magnetic resonance imaging indicates that this was primarily driven by a loss of fat mass. Further, from the estimated plasma half-life in rats, dogs, and minipigs, it could be anticipated that NN1213 would have a PK profile compatible with once-weekly subcutaneous administration in humans. Thus, NN1213 is an interesting tool compound for elucidating the effects from the AMYR and CTR, respectively, that could also be an attractive candidate for further clinical development as an antiobesity treatment.

## Experimental Section

### Chemistry

#### Synthesis

The synthesis of peptides was performed on Prelude and Liberty commercial peptide synthesizers using the manufacturer’s protocols for Fmoc peptide synthesis using standard protected amino acids. The coupling reagent was OxymaPure/DIC, and the resin was Rink amide resin. Piperidine 25% was used to remove the Fmoc group, and coupling was done by using 0.3 M Fmoc-amino acid in 0.3 M OxymaPure in DMF added in 6–8 equiv excess and activated by 6–8 equiv of DIC (3 M solution in DMF) and 3–4 equiv of collidine (3 M solution in DMF). Coupling time was typically 30–60 min at room temperature on the Prelude. Cysteine was trityl-protected, and the formation of the disulfide bridge was performed on resin by treatment with a solution of iodine (detailed methods are described in Kruse et al.^7^). Cleavage was done with 95% trifluoroacetyl (TFA), 2.5% water, and 2.5% triisopropylsilane for up to 3 h, followed by diethyl ether precipitation and preparative HPLC on C18 reverse phase columns using 0.1% TFA in water and acetonitrile as eluents, followed by lyophilization. Purity of all compounds was established by HPLC/UPLC and identity was confirmed by LCMS/MALDI-TOF. All compounds were confirmed to be ≥95% pure by the HPLC/UPLC analysis. See Supporting Information S6: Materials and Methods of Peptide Synthesis.

#### Solubility versus pH Profiles

The solubility profile was assessed by mixing 50 μL aliquots of a 500 μM aqueous solution of each compound with 50 μL of 100 mM pH-adjusted buffer solutions (lactate pH 3–5; bis-tris-propane pH 6–8) to a nominal concentration of 250 μM. Samples were left overnight at room temperature to reach solubility equilibrium and subsequently centrifuged to isolate the supernatant. Peptide concentration in the supernatant was determined by ultraperformance liquid chromatography (UPLC) using an Acquity UPLC column (bridged ethylsiloxane/silica hybrid C18 1.7 μM – 2.1 × 50 mm) with a flow rate of 0.45 mL/min at 40 °C and detection at 214 nm. A gradient combining eluent A (0.05% TFA in water) and eluent B (0.05% TFA in acetonitrile) was applied (% A/% B: 0 min: 95/5; 0–0.5 min: linear to 90/10; 0.5–2.5 min: linear to 35/65; 2.5–3 min: linear 0/100; 3–4 min: 0/100; 4–4.5 min: linear to 95/5; 5 min: 95/5). Measured values ≥200 μM were reported as “>200 μM”, whereas measured values below 200 μM were reported as is. Calculation of pI was performed using ACDLabs software.

#### Compound Stability

The stability properties of the individual amylin analogs were assessed with respect to their propensity toward amyloid fibril formation. The relative amount of covalent dimers and polymers (HMWP) present prior to fibril formation testing was measured as a control for the potential inhibitory effect of HMWP on fibril formation propensity as reported elsewhere.^39^

#### Tests for Fibril Formation Propensity (ThT Assay)

The stability properties of the individual amylin analogs were assessed with respect to their propensity toward fibril formation, expressed as the time until fibril formation (measured as Lag time) and loss of dissolved peptide (assessed as Peptide Recovery). The propensity toward formation of fibrils upon exposure to mechanical stress was assessed as previously described for insulin formulations^40^ using the thiazole dye ThT, which exhibits specific fluorescence characteristics in the presence of amyloid fibrils. Samples were prepared as described previously (using the same methodology and materials) for a novel amylin-based analog candidate compound.^7^

Briefly, each compound was dissolved in 10 mM of glycylglycine buffer (pH 4.0) or HEPES buffer (pH 7.5) to a concentration of 250 μM, followed by ThT stock solution to a final concentration of 1 μM. Each solution was aliquoted into a microtiter plate (200 μL/well) sealed with a transparent adhesive sheet. Mechanical stress was applied at 37 °C incubation (960 rpm, 1 mm amplitude) with 20 min fluorescence reading intervals for 45 h (filters: excitation 444 nm; emission 485 nm). Each peptide was tested in experiments consisting of four replicas (e.g., four wells on the same plate), and the lag time of each replica was determined by visual inspection of the fluorescence-versus-time plot. For wells where no fibril formation could be detected, the lag time was set longer than the test period (>45 h). The lag time of the peptide was set to the lowest lag time of the four replicates (conservative estimate). Some of the peptides were tested in more than one experiment, and the lag time results given in Tables 6 and 9 represent the average of these experiments. The ThT time course data given in Supporting Information represent the average and SEM of each experiment.^40^ After analysis, wells representing individual replicas were pooled, and the residual soluble peptide was isolated using centrifugation (20,000 *g*/30 min at room temperature) followed by 0.22 μM filtration. The amount of residual dissolved peptide was determined using an HPLC with a flow rate of 2 mL/min at 30 °C and 215/276 nm detection. A gradient combining eluent A (7.7% w/w acetonitrile, 200 mM Na_2_SO_4_, 20 mM NaH_2_PO_4_, 20 mM Na_2_HPO_4_, pH 7.2) and eluent B (65.5% w/w acetonitrile) was applied. The amount of residual dissolved peptide was reported on a relative scale (Peptide Recovery) to the amount of dissolved peptide prior to mechanical stress.

#### Assessment of High Molecular Weight Product Content by Size-Exclusion Chromatography

The content of HMWP (sum of covalent dimer and polymers) was determined using a Waters Alliance 2695 HPLC system equipped with a Waters Insulin HMWP column (7.8 × 300 mm; Waters Corp., Milford, MA) with a flow rate of 0.5 mL/min at 50 °C and detection at 215 nm. Elution was performed under isocratic conditions with the following mobile phase prepared in Milli-Q water (Millipore A/S): 0.5 M NaCl, 10 mM sodium dihydrogen monohydrate, 5 mM ortho-phosphoric acid, 50% (v/v) 2-propanol. The amount of HMWP was determined as the absorbance area of the corresponding peaks given in percentage of the total absorbance area. The molar extinction coefficients of the molecules eluting in the HMWP fraction and the main peak was assumed to be equal.

### In Vitro Assays

#### Screening Assays for SAR Analysis

##### Functional Analysis

The potency of the peptides against the human AMY_3_R and CTRs or the rat AMY_3_R or CTR was measured in a cAMP-responsive element luciferase reporter assay performed on baby hamster kidney cells stably expressing the reporter and the designated receptors. For the luciferase assay, cells were thawed the day before the experiment and incubated overnight. On the day of the experiment, cells were washed and incubated for 3 h with the peptide. The medium was removed and replaced by phosphate-buffered saline and SteadyLite in a 1:1 ratio and incubated at room temperature for 30 min before luminescence was measured. In the cAMP assay, the transfected cells were thawed and seeded into FlashPlates on the day of the experiment and incubated with peptide for 30 min. The reaction was stopped with a detection mix. The plates were then sealed with plastic, shaken, and allowed to stand at least 2 h before scintillation was measured. EC_50_ values were calculated in GraphPad Prism. See Supporting Information S7: SAR Screening Assay Methods (Functional Assays (Human and Rat)) for more information.

##### Binding Analysis

The apparent binding affinities were determined using a scintillation proximity assay with beads from PerkinElmer and cell membranes containing the AMY_3_R or CTRs. Baby hamster kidney cells transiently expressing the human or rat receptor were cultured for 24 h. Membranes were harvested and membranes were prepared and kept at −80 °C until use. The assay was performed in a 384-well Optiplate (PerkinElmer). Membranes were mixed with scintillation proximity assay beads in a 1:1 ratio. Peptides were dissolved in DMSO and further diluted in assay buffer and added to the Optiplate together with the dissolved radioligand. ^125^I-Calcitonin (NEX422 PerkinElmer) and ^125^I-rat amylin (NEX448 PerkinElmer) were used as radioligands in the CTR and AMY_3_R assays, respectively. The final mixture was incubated for 120 min at 25 °C prior to centrifugation. Samples were analyzed on a TopCounter (Packard). The IC_50_ values were calculated using GraphPad Prism. See Supporting Information S7: SAR Screening Assay Methods (Binding Assays (Human and Rat)) for more information.

#### Functional Human Calcitonin Family Receptor Assays (BacMam)

For further in vitro characterization of the peptides, the baculovirus gene transfer into mammalian cells (BacMam) system was used to transduce HeLa cells with the full range of human calcitonin family receptors, and the cells were stimulated with NN1213, cagrilintide, sCT, pramlintide, or other endogenous calcitonin family peptides. Cagrilintide, pramlintide, and sCT were included as reference compounds for comparison, and native endogenous ligands were included as pharmacological tools to measure changes in receptor functionality following the coexpression of CTR or CRLR with RAMPs. Stimulation of calcitonin family receptors activates adenylyl cyclase, leading to accumulation of downstream second messenger cAMP when 3-isobutyl-1-methylxanthine is added. Increasing levels of endogenous cAMP were measured as a reduction in fluorescence resonance energy transfer between Europium (Eu3^+^)-cryptate-conjugated anti-cAMP antibody and d2-conjugated cAMP. The fluorescence ratio was plotted as a function of the concentration of the compound. Outliers were identified and removed by the robust regression and outlier removal method,^41^ and the cleaned data were analyzed by nonlinear curve fitting of a three-parameter logistic function with shared top and bottom using GraphPad Prism software (version 8.0.2). See Supporting Information S1: BacMam Functional Assay Methods (Human) for further information.

### In Vivo Models

In vivo experiments were approved under a license from the Danish Animal Experiments Inspectorate. All animals had access to shelter, nesting material, and chewing sticks and were acclimatized and getting used to handling at least 1 week prior to any experiments. Rats were housed in groups, except for 1 week prior to and during the monitoring of the food intake.

#### Pharmacokinetics

PK studies were conducted in rats, dogs, minipigs, and rabbits. Briefly, animals were dosed with a single sc or iv dose of NN1213. Blood samples were collected at fixed time points postdose to determine full PK profiles. Plasma samples were analyzed by ELISA. Plasma concentration–time profiles were analyzed by noncompartmental analysis. Further details are provided in Supporting Information S5: In vivo Methods (Pharmacokinetic Evaluation).

#### Acute Food Intake in Rats and Dogs

Food intake was measured in rats (*n* = 5–7 per group) for a period of up to 48 h after a single sc injection of the peptide. Food intake was measured in dogs (*n* = 3–9 per group). For dogs, food intake (four meals) was measured over 48 h. In the rat study, the inhibition of food intake was calculated from the mean accumulated amount of food eaten compared to the amount of food eaten by a rat in the control group per 0 to 24 h or 24 to 48 h. In this way, not only the magnitude of the effect but also the duration was comparable across the screened peptides. Further details are provided in Supporting Information S5: In vivo Methods (Efficacy Evaluation).

#### Sub-chronic Weight Loss in Rats

The ability of NN1213 to reduce body weight was tested in DIO rats fed an HFD. Rats were dosed once daily sc with either vehicle or NN1213 (*n* = 8–10 per group). Food intake and body weight were measured daily on individually housed rats. Body composition (magnetic resonance scan) was measured 6 days prior to the first dose and at day 21. Body fat and lean mass are expressed as changes from baseline. Further details are provided in Supporting Information S5: In vivo Methods (Efficacy Evaluation).

## Supporting Information Available

Additional experimental methods, associated results for in vitro assays and in vivo evaluations, and methods for peptide synthesis (PDF)

## Abbreviations

AM1: adrenomedullin 1
AM_1_R: AM1 receptor
AM2: adrenomedullin 2
AM_2_R: AM2 receptor
AMR: adrenomedullin receptor
AMY_1_R: amylin-1 receptor
AMY_2_R: amylin-2 receptor
AMY_3_R: amylin-3 receptor
AMYR: amylin receptor
BacMam system: baculovirus gene transfer into mammalian cells
CGRPR: calcitonin gene-related peptide receptor
CI: confidence interval
CRLR: calcitonin receptor-like receptor
CTR: calcitonin receptor
hAM_1_R: human AM_1_R
hAM_2_R: human AM_2_R
hAMY_1_R: human AMY_1_R
hAMY_2_R: human AMY_2_R
hAMY_3_R: human AMY_3_R
hCGRPR: human CGRPR
hCTR: human CTR
HeLa: Henrietta Lacks
HFD: high fat diet
HMWP: high molecular weight product
NA: no activity
ND: not done
pEC_50_: negative logarithm of the EC_50_
pI: isoelectric point
pIC_50_: negative logarithm of the IC_50_
RAMP: receptor activity-modifying protein
rAMY_3_R: rat AMY_3_R
rCTR: rat CTR
sCT: salmon calcitonin
SD: standard deviation
SEM: standard error of the mean
TFA: trifluoroacetyl
ThT: thioflavin T
UPLC: ultraperformance liquid chromatography
