# Correction to “NN1213 – A Potent, Long-Acting, and Selective Analog of Human Amylin”

**DOI:** 10.1021/acs.jmedchem.4c02359

**Published:** 2024-12-12

**Authors:** Kirsten Dahl, Kirsten Raun, Jakob Lerche Hansen, Christian Poulsen, Charlotta D. de la Cour, Trine Ryberg Clausen, Ann Maria Kruse Hansen, Linu M. John, Annette Plesner, Gao Sun, Morten Schlein, Rikke Bjerring Skyggebjerg, Thomas Kruse

*Correction*: The following paragraph previously on page 11691 is now the footnote for Table 12 on page 11693.

“Data are mean ± SEM measured as percent food intake relative to vehicle in rats after a single sc injection of 30 nmol/kg of the test substance. Data for cagrilintide have been published previously^7^ giving rise to 85% and 84% reduction of food intake in the periods from 0–24 h and 24–48 h, respectively. SEM is the standard error of the mean.”

*Correction:* The following paragraph was previously included as the footnotes for Table 9 on page 11692 and has been moved to page 11691.

“From the in vitro data (Tables 10 and 11), it was apparent that selectivity was achieved for several peptides (**10**, **14**, **15**, **16**, **17**, **18**, **20**, and **21**). From the ThT assay (Table 9 and Supporting Information), only three of the AMYR selective peptides (**14**, **16**, and **21**) were able to resist physical stress for more than 40 h. Two peptides (**15** and **21**) had a selectivity ratio above 30. Peptide 6 and peptides **11**–**21** with neutral/high pI were chosen for further evaluation in vivo to evaluate their ability to reduce food intake in rats after a single administration and to have increased insights into the duration of effects from the peptides in vivo (Table 11).”

