# Supplementary material for: NN1213 – A Potent, Long-Acting, and Selective Analog of Human Amylin
Source: J Med Chem. 2024 Jul 3;67(14):11688–700. doi: 10.1021/acs.jmedchem.4c00022 (PMC11284788; doi:10.1021/acs.jmedchem.4c00022)
Supplement: Supplementary file 1 — jm4c00022_si_001.pdf [file jm4c00022_si_001.pdf]

Supporting Information

NN1213 – A Potent, Long-Acting, and Selective Analog of Human Amylin

Kirsten Dahl[a], Kirsten Raun[a], Jakob Lerche Hansen[a†], Christian Poulsen[a], Charlotta D. de la Cour[a], Trine Ryberg Clausen[a], Ann Maria Kruse Hansen[a], Linu M. John[a,b‡], Annette Plesner[a§], Gao Sun[b‖], Morten Schlein[a¶], Rikke Bjerring Skyggebjerg[a], and Thomas Kruse[a]*

[a]Novo Nordisk A/S, Novo Nordisk Park, DK-2760 Maaloev, Denmark

[b]Novo Nordisk China, Novo Nordisk Research Center China, Building 2, 20 Life Science Park Road, Changping District, 102206 Beijing, China

*Email: KDHL@novonordisk.com

Present Addresses:

†Blue Cell Therapeutics, Ole Maaløes Vej 3, DK-2200 Copenhagen, Denmark

‡Global Health, Bill & Melinda Gates Foundation, 500 5th Avenue, Seattle, Washington 98104, United States

§Copenhagen University Hospital, Ole Maaløes Vej 26, DK-2200 Copenhagen N, Denmark

‖PegBio Co. Ltd., BioBay Building A3 (Suite 418), 218 Xinghu Street, Suzhou Industrial Park, Suzhou, 215123 Jiangsu Province, China

¶Gubra A/S, Hørsholm Kongevej 11B, DK-2970 Hørsholm, Denmark

TABLE OF CONTENTS

S1: BacMam Functional Assay Methods (Human). 2
S2: BacMam Functional Assay Results (Human). 6
S3: Species-Specific *In vitro* Assay Methods........ 7
S4: Species-Specific *In vitro* Assay Results. 9
S5: *In vivo* Methods. 11
S6: *In vivo* Results. 14
S7: Materials and Methods of Peptide Synthesis. 15
S8: SAR Screening Assay Methods. 20
S9: SAR Screening Assay Results........ 24
S10: ThT Fluorescence Time Courses........ 27
REFERENCES 29

S1: BacMam Functional Assay Methods (Human).

The baculovirus gene transfer into mammalian cells (BacMam system) was used to transduce Henrietta Lacks (HeLa) cells with the full range of human calcitonin family receptors (Table S1), and the cells were stimulated with NN1213 (i.e., peptide **21**), salmon calcitonin (sCT), pramlintide, or endogenous calcitonin family peptides. Pramlintide and sCT were included as reference compounds for comparison, and native endogenous ligands were included as pharmacological tools to measure changes in receptor functionality following co-expression with receptor activity-modifying proteins (RAMPs). Stimulation of calcitonin family receptors activates adenylyl cyclase, leading to accumulation of downstream second messenger cAMP when 3-isobutyl-1-methylxanthine (IBMX) is added. Increasing levels of endogenous cAMP were measured as a reduction in fluorescence resonance energy transfer (FRET) between Europium ($Eu3^{+}$)-cryptate-conjugated anti-cAMP antibody and d2-conjugated cAMP. The fluorescence ratio was plotted as a function of the concentration of the compound. Outliers were identified and removed by the robust regression and outlier removal (ROUT) method (Motulsky et al.[1]), and the cleaned data were analyzed by nonlinear curve fitting of a three-parameter logistic function with shared top and bottom using GraphPad Prism.

Table S1. Calcitonin Family Receptors Used in BacMam Assays

Abbreviation	Receptor name	GPCR	RAMP subtype
hCTR	Human calcitonin receptor	$hCTR_a$	-
$hAMY_1R$	Human amylin-1 receptor	$hCTR_a$	hRAMP1
$hAMY_2R$	Human amylin-2 receptor	$hCTR_a$	hRAMP2
$hAMY_3R$	Human amylin-3 receptor	$hCTR_a$	hRAMP3
hCRLR	Human calcitonin receptor-like receptor	hCRLR	-
hCGRPR	Human calcitonin gene-related peptide receptor	hCRLR	hRAMP1
hAM_1R	Human adrenomedullin 1 receptor	hCRLR	hRAMP2
hAM_2R	Human adrenomedullin 2 receptor	hCRLR	hRAMP3

GPCR, G-protein coupled receptor; hRAMP, human RAMP.

Test Substances. Test substances (Table S2) were formulated in stock solutions of 25 μM or 50 μM in 0.1 M acetic acid (sCT, pramlintide, and NN compounds) or 50 μM in 80% DMSO (reference compounds).

Table S2. Test Substance and Stock Solutions Used in BacMam Assays

Compound	Description	Amount (μM)	Buffer	Source
NN1213	Peptide 21	25	0.1 M acetic acid	NN1213 batch 11K, Novo Nordisk A/S)
Cagrilintide	Peptide 23	25	0.1 M acetic acid	NN833 batch 16A, Novo Nordisk A/S)
Pramlintide	-	25	0.1 M acetic acid	ChemPep Inc., Wellington, FL, US
sCT	Salmon calcitonin	25 or 50	0.1 M acetic acid	T3660, Sigma-Aldrich, St. Louis, MO, US
hCT	Human calcitonin	50	80% DMSO	H-2250, Bachem, Bubendorf, Schweiz
hβ-CGRP	Human β-calcitonin gene-related peptide	50	80% DMSO	C0167, Sigma-Aldrich, St. Louis, MO, US
hAMY	Human amylin	50	80% DMSO	H-7905, Bachem, Bubendorf, Schweiz
r/mAMY	Rat or mouse amylin	50	80% DMSO	H-9475, Bachem, Bubendorf, Schweiz
hAM1	Human adrenomedullin 1	50	80% DMSO	H-2932, Bachem, Bubendorf, Schweiz
hAM2	Human adrenomedullin 2	50	80% DMSO	H-6064, Bachem, Bubendorf, Schweiz

Principle. BacMam viruses are modified baculoviruses that contain mammalian expression cassettes for viral gene delivery and transient expression in mammalian cells. The controlled titrate-able nature of the BacMam system is particularly useful for the double transductions needed when working with the complex pharmacology of the calcitonin family receptors.

Calcitonin receptor (CTR) and calcitonin receptor-like receptor (CRLR) are both stimulatory G-protein (G_s)-coupled 7 transmembrane receptors. Accumulation of cAMP following stimulation of the receptors was measured using the cAMP G_s Dynamic Homogeneous Time Resolved Fluorescence (HTRF) assay kit, which was commercially available from CisBio Bioassays, France (Cat# 62AM4PEC). For these experiments, lot# 11E (experiment 1) and 11A (experiments 2 and 3) were used. The method is based on a monoclonal antibody specific for cAMP, labelled with $Eu3^+$-cryptate. This antibody competes with native cAMP produced by cells and cAMP coupled to the dye d2. The specific signal is inversely proportional to the concentration of cAMP in the cell lysate. Data reduction using the fluorescence ratio (10^4 x 665 nm /620 nm, i.e., 10^4 times the fluorescence at 665 nm divided by the fluorescence at 620 nm) eliminates possible photophysical interference and means the assay is unaffected by buffer conditions and colored compounds.

Cell Culture. HeLa cells were cultured in an incubator (95% relative humidity [RH], 5% [of standard atmospheric pressure] CO_2, 37 °C) in cell culture medium (Dulbecco's Modified Eagle Medium [DMEM] with 10% [v/v] fetal bovine serum [FBS], and 1% [w/w] penicillin/streptomycin).

Cell Plating in T75 Flasks (Day 1). The day before cell transduction, 2 x 10^6 HeLa cells were seeded in nine T75 flasks. Media was removed from 2–3 T175 flasks, and cells were washed once with phosphate-buffered saline PBS (with Mg^{2+} and Ca^{2+}). Cells were loosened with 2 mL TrypLE for 5 min in an incubator. Five mL pre-warmed media were added to each T175 flask, mixed with the cell suspension, and transferred (gather cells from all T175 flasks)

to one 50 mL NUNC tube. The cell suspension was centrifuged at 1200 rpm for 4 min, the supernatant was discarded, and cells were reconstituted in 10 mL media. Cells were counted in 100 µL of this suspension. The cell suspension was centrifuged at 1200 rpm for 4 min and the supernatant was discarded. The cells were re-suspended in media and 2 x 10^6 cells were seeded in approximately 15 mL in each of the nine T75 flasks. The flasks were kept overnight in an incubator (95% RH, 5% CO_2, 37 °C).

Transduction of Cells (Day 2). Reagents were acclimated to room temperature for 30 min. Media was aspirated from the T75 flasks and cells were washed once with PBS (with Mg^{2+} and Ca^{2+}). Cells were transduced for 3 h with the appropriate receptor expression bacmids in 4 mL PBS (with Mg^{2+} and Ca^{2+}), as in Table S3.

Table S3. Human Receptor Expression Bacmids

Cells	GPCR bacmid	RAMP bacmid
Non-transfected HeLa cells	-	-
hCTR	5% (v/v) hCTR	-
$hAMY_1R$	5% (v/v) hCTR	15% (v/v) hRAMP1
$hAMY_2R$	5% (v/v) hCTR	15% (v/v) hRAMP2
$hAMY_3R$	5% (v/v) hCTR	15% (v/v) hRAMP3
hCRLR	5% (v/v) hCRLR	-
hCGRPR	5% (v/v) hCRLR	15% (v/v) hRAMP1
hAM_1R	5% (v/v) hCRLR	15% (v/v) hRAMP2
hAM_2R	5% (v/v) hCRLR	15% (v/v) hRAMP3

Seeding of Transfected Cells in Assay Plates (Day 2). Following the 3-h transduction, T75 flasks were washed once with PBS (with Mg^{2+} and Ca^{2+}). The cells were detached with 1 mL TrypLE and washed once in media. After discarding the supernatant, the cells were re-suspended in 5 mL pre-warmed media and counted. The cell suspension was centrifuged at 1200 rpm for 4 min and the supernatant was discarded. Then, cells were re-suspended in media to a cell density of 3 x 10^5 cells/mL before being seeded with 1.2 x 10^4 cells/well (40 µL) in poly-d-lysine coated 384-well assay plates. The plates received a short spin at 1300–1500 rpm and were then incubated overnight in a CO_2-permeable plastic bag.

cAMP HTRF Assay (Day 3). On the day of the experiment, media was removed from the assay plates and the cells were washed twice with 40 µL DMEM w/o phenol red using a BioMek NX assay robot (Beckman coulter life sciences, Indianapolis, IN, US). Fifteen µL of assay buffer (consisting of DMEM w/o phenol red, 10 mM HEPES, 1x Glutamine, 1% [w/w] penicillin/streptomycin, 0.1% ovalbumin, and 1.5 mM IBMX, pH 7.4) was added to all wells using a BioMek NX assay robot. Subsequently, 15 µL of test or reference compounds in serial 10-fold dilutions from 10^{-6} M to 10^{-12} M in assay buffer was added (using technical duplicates, i.e., two wells of each sample, one data point being the average of two wells). Plates were spun at 1300–1500 rpm and incubated for 30 min at room temperature followed by the addition of 6 µL each of d2-conjugated cAMP and Eu^{3+}-cryptate-conjugated anti-cAMP antibody in lysis buffer (lysis buffer, d2-conjugated cAMP and Eu^{3+}-cryptate-conjugated anti-cAMP antibody were all included with the cAMP HTRF Assay kit referred to above). The plates were spun at 1300–1500 rpm and incubated for 1 h at room temperature before reading on a Mithras LB 940 fluorescence plate reader (Berthold Technologies, Bad Wildbad, Germany) using filters for excitation at 320 nm and emissions at 665 nm and 620 nm. Increasing levels of endogenous cAMP were measured as a reduction in FRET between $Eu3^+$-cryptate-conjugated anti-cAMP antibody and d2-conjugated cAMP. Data from the

fluorescence reader was given as the dimensionless fluorescence ratio (10^4 x 665 nm /620 nm).

Data Analysis. The fluorescence ratio was plotted as a function of the concentration of the compound. Outliers were identified and removed by the ROUT method, and the cleaned data were analyzed by nonlinear curve fitting of a three-parameter logistic function with shared top and bottom using GraphPad Prism. Concentrations giving EC_{50} were estimated from the dose-response curves and converted to pEC_{50}values (–log EC_{50}). All experiments were performed with technical duplicates and repeated three times. Calculations of mean pEC_{50} values and lower and upper 95% confidence intervals (CIs) were performed in GraphPad and data are reported with two digits.

S2: BacMam Functional Assay Results (Human).

Table S4. *In vitro* Potencies on Calcitonin Family Receptors in BacMam Assay

	pEC_{50} (95% CI)						
Peptide	CTR	AMY_1R	AMY_2R	AMY_3R	CGRPR	AM_1R	AM_2R
NN1213	8.76 (7.87–9.65)	10.24 (9.55–10.93)	9.74 (8.64–10.84)	10.12 (9.23–11.00)	6.57 (6.24–6.90)	NA	NA
Cagrilintide	10.64 (10.10-11.18)	10.73 (9.97-11.50)	10.37 (9.77-10.97)	10.33 (9.92-10.74)	6.25 (5.87[a] -6.63)	NA	6.12 (5.85[a] -6.39)
Pramlintide	9.48 (8.16–10.80)	10.66 (9.53–11.80)	10.19 (8.65–11.74)	10.53 (9.18–11.89)	7.63 (6.78–8.49)	NA	6.83 (6.15–7.52)
sCT	11.12 (11.01–11.23)	11.21 (10.78–11.65)	11.26 (10.83–11.69)	10.87 (10.33–11.40)	6.41 (5.90[a]–6.92)	NA	NA
hCT	10.53 (10.37–10.70)	10.51 (10.14–10.89)	10.51 (9.93–11.09)	8.89 (8.23–9.55)	6.94 (6.19–7.69)	NA	NA
hAMY	8.70 (7.58–9.82)	10.01 (9.66–10.36)	9.34 (8.48–10.20)	9.78 (8.96–10.60)	7.08 (6.81–7.34)	NA	6.35 (6.25–6.45)
r/mAMY	8.68 (7.78–9.59)	10.12 (9.30–10.93)	9.57 (8.47–10.67)	9.81 (8.78–10.83)	7.52 (7.16–7.89)	NA	6.92 (6.82–7.01)
hβ-CGRP	8.30 (7.21–9.39)	10.50 (9.48–11.52)	9.49 (7.77–11.22)	9.37 (7.21–11.52)	10.32 (9.62–11.01)	8.28 (7.60–8.96)	7.92 (7.66–8.19)
hAM1	7.35 (6.75–7.95)	8.09 (7.50–8.68)	8.57 (7.73–9.41)	7.71 (7.10–8.32)	8.11 (7.78–8.44)	9.08 (8.80–9.37)	9.13 (8.88–9.39)
hAM/AM2	7.22 (6.68–7.77)	8.91 (8.28–9.54)	7.73 (7.07–8.38)	8.01 (7.21–8.81)	8.79 (8.39–9.19)	8.19 (7.87–8.51)	9.34 (9.11–9.57)

[a]Data below six are extrapolated out of range for test concentrations. n = 3 for CTR, AMY_1R, AMY_2R, and AMY_3R. n = 4 for CGRPR, AM_1R, and AM_2R. AM_1R, AM1 receptor; AM_2R, AM2 receptor; AMY_1R, amylin-1 receptor; AMY_2R, amylin-2 receptor; AMY_3R, amylin-3 receptor; CGRPR, calcitonin gene-related peptide receptor; NA, no activity.

S3: Species-Specific *In vitro* Assay Methods.

Human, Rat, and Canine Receptors. *Transient transfections:* To analyze CTR, AMY_1R, and AMY_3R potencies, and binding affinities, baby hamster kidney (BHK) tk-ts 13 cells were transiently transfected with the CTR or with the CTR and RAMP1/3 in a ratio (1:3) for the species of interest. Transfections were performed using FuGENE 6 (Roche), according to the manufacturer's recommendations. Cells were grown in DMEM with 10% FBS and 1% penicillin/streptomycin. The next day, the cells were handled as described below.

cAMP assay (human, rat, canine receptors): Approximately 24 h after transfection (with human, rat, or canine receptors) the cells were detached, counted, and re-suspended at the required density in DMEM with 10% FBS and 0.1% penicillin/streptomycin. The cells were then transferred to a 384-well plate (10000 cells/well in a volume of 5 µL). Then 5 µL of the analogs of interest were added (2x concentration) in assay buffer (4-fold dilution series 4*10e^-8 – 4*10e^-15 (final concentration), last dilution contains no compound). The plates were centrifuged briefly at 1500 rpm and incubated for 30 min at room temperature. Next, 5 µL cAMP-d2 in lysis/conjugate buffer and 5 µL Cryptate was added, and the plates were centrifuged briefly at 1500 rpm and incubated for 60 min at room temperature and then read on Mithras (program HTRF384). cAMP was quantitated using a standard curve and curves plotted in GraphPad Prism. EC_{50} values were calculated in GraphPad Prism using a nonlinear regression with hillslope=1.

Whole-cell competition binding (human, rat, canine receptors): Approximately 24 h after transfection (with either human, rat, or canine versions of CTR, AMY_1R, or AMY_3R), cells were seeded into Poly-D-Lysin coated 384W Opaque White, BD BioCoat plates (10000 cells/well) and incubated overnight at 37 °C, 5% CO_2, 95% humidity. Cells were then washed in Hank's Balanced Salt Solution (HBSS) (4 °C) and incubated overnight at 4 °C in a binding buffer containing test compounds (50 pM [^{125}I]-human calcitonin [for CTR-only expressing cells] or 50 pM [^{125}I]-rat amylin [for AMY_1R- and AMY_3R-expressing cells] in Dulbecco media w/o phenol red, 0.1% ovalbumin, 10 mM HEPES, 1x Glutamine, 1% P/S, and 0.1% Pluronic F68). The following morning, the plates were washed three times in HBSS (4 °C) and lyzed in Lysis buffer (0,1 M sodium hydroxide [VWR #1.09136.1000]), 1% sodium dodecyl sulfate. Then MicroScint40 was added and the plates were briefly shaken at 500 rpm. Subsequently, the plate was incubated at room temperature in the dark for 1 h and read on a TopCounter. The IC_{50} was calculated using (one site binding competition analysis) GraphPad Prism5 as a measure of receptor affinity.

Mouse Receptors (Functional Assay Only). *Cell culture:* HeLa cells were cultured in an incubator at 37 °C and 5% CO_2, in cell culture medium (DMEM with 10% [v/v] FBS, 1% [w/w] penicillin/streptomycin).

Cell seeding in T75 flasks (day 1): The day before transfection of the cells, 2 x 10^6 HeLa cells were seeded in three T75 flasks and incubated overnight. Media was removed from 1–2 T175 flasks and cells were washed once with Dulbecco PBS (with Mg^{2+} and Ca^{2+}). Cells were detached with 2 mL TrypLE Express for 5 min in an incubator. Five mL pre-warmed media was added to each T75 flask and mixed with the cell suspension. Cells were counted in 100 µL of this suspension. The cell suspension was centrifuged at 1200 rpm for 4 min and the supernatant was discarded. The cells were re-suspended in media and 2 x 10^6 cells were seeded in approximately 15 mL in each of the three T75 flasks. The flasks were incubated overnight.

Coating of assay plates with poly-d-lysin (day 2): Small volume 384 assay plates were incubated with 20 µL poly-d-lysin/well for 2–3 h at 37 ºC. The plates were washed twice with PBS and allowed to dry.

Transfection of cells (day 2): Reagents were acclimated to room temperature for 30 min. Media was removed from the T75 flasks, and cells were washed once with PBS (with Mg^{2+} and Ca^{2+}). Cells were transduced for 3 h (95% RH, 5% CO_2, 37 °C) with the appropriate receptor expression bacmids in 4 mL PBS (with Mg^{2+} and Ca^{2+}) as shown in Table S5.

Table S5. Mouse Receptor Expression Bacmids

Cells	GPCR bacmid	RAMP bacmid
Non-transfected HeLa cells	-	-
$mCTR_{(a)}$	1% (v/v) mCTR	-
$mAMY_3R_{(a)}$	1% (v/v) mCTR	1% (v/v) mRAMP3

$mAMY_3R$, mouse AMY_3R; mCTR, mouse CTR; mRAMP, mouse RAMP.

Seeding of transfected cells in assay plates (day 2): Following the 3-h transduction, T75 flasks were washed once with PBS (with Mg^{2+} and Ca^{2+}). The cells were detached with 1 mL TrypLE Express and washed once in cell culture media. After discarding the supernatant, the cells were re-suspended in 5 mL pre-warmed media and counted. The cell suspension was centrifuged at 1200 rpm for 4 min and the supernatant was discarded. Then, cells were re-suspended in media to a cell density of 3 x 10^5 cells/mL before being seeded with 6 x 10^3 cells/well (20 µL) in the poly-d-lysin coated 384-well assay plates. The plates were briefly spun at 1300–1500 rpm and were then placed overnight in an incubator (37 °C, 5% CO_2).

cAMP HTRF assay (day 3): On the day of the experiment, media was removed from the assay plates and the cells were washed twice with 20 µL assay buffer (consisting of DMEM w/o phenol red, 10 mM HEPES, 1x Glutamine, 1% [w/w] penicillin/streptomycin, 0.1% ovalbumin, 1.5 mM IBMX, pH 7.4). Ten µL of test or reference compounds in serial 4-fold dilutions in assay buffer was added (using technical duplicates, i.e., two wells of each sample, one data point being the average of two wells). Each dilution series contained eleven dilutions and a blank. The starting concentration was 10^{-6} M for cagrilintide and pramlintide and 100-fold lower at 10^{-8} M for sCT. Plates were incubated for 30 min at room temperature, followed by the addition of 5 µL each of d2-conjugated cAMP and Eu^{3+}-cryptate-conjugated anti-cAMP antibody in lysis buffer (lysis buffer, d2-conjugated cAMP and Eu^{3+}-cryptate-conjugated anti-cAMP antibody were all included with the cAMP HTRF Assay kit referred to above). The plates were incubated for 1 h at room temperature before being read on a Mithras LB 940 fluorescence plate reader using filters for excitation at 320 nm and emissions at 665 nm and 620 nm. Increasing levels of endogenous cAMP were measured as a reduction in FRET between $Eu3^{+}$-cryptate-conjugated anti-cAMP antibody and d2-conjugated cAMP. Dimensionless data from the fluorescence reader was given as the fluorescence ratio (10^4 x 665 nm/620 nm).

Data analysis: The fluorescence ratio was plotted as a function of the concentration of the compound. All data were plotted and analyzed in GraphPad Prism by nonlinear curve fitting of a four-parameter logistic function. pEC_{50} values (–log EC_{50}) were derived from the dose-response curves along with lower and upper 95% CIs for the five experiments.

S4: Species-Specific *In vitro* Assay Results.

To evaluate our peptide analogs in more detail and to determine if mice, rats, and canines are suitable models for measuring the efficacy of our compounds, the binding affinities (Table S6) and functional potencies (Table S7) of NN1213/peptide **21**, pramlintide, and sCT were compared in human cell lines transiently transfected with the human, mouse, rat, or canine CTR, AMY_1R, or AMY_3R. We were not successful in developing a robust binding assay for the mouse receptors; thus, we have only reported data for the functional potencies.

Table S6. Species-Specific Binding Affinities on CTR, AMY_1R, and AMY_3R

	$hAMY_1R$			$hAMY_3R$			hCTR			Ratio	
	pIC_{50}	(95% CI)	*N*	pIC_{50}	(95% CI)	*N*	pIC_{50}	(95% CI)	*N*	CTR/AMY_1R	CTR/AMY_3R
Pramlintide	9.40	0.13	4	9.46	0.18	4	7.33	0.20	4	117	134
sCT	9.41	0.06	4	9.68	0.05	4	9.64	0.05	4	1	1
NN1213	8.65	0.15	4	8.40	0.20	4	6.57	0.45	4	120	67
	$cAMY_1R$			$cAMY_3R$			cCTR			Ratio	
	pIC_{50}	(95% CI)	*N*	pIC_{50}	(95% CI)	*N*	pIC_{50}	(95% CI)	*N*	CTR/AMY_1R	CTR/AMY_3R
Pramlintide	9.07	0.26	4	9.29	0.19	4	7.24	0.24	4	67	112
sCT	9.31	0.12	4	9.52	0.39	4	9.20	0.15	4	1	2
NN1213	8.31	0.20	4	8.04	0.28	4	6.44	0.40	4	73	40
	$rAMY_1R$			$rAMY_3R$			rCTR			Ratio	
	pIC_{50}	(95% CI)	*N*	pIC_{50}	(95% CI)	*N*	pIC_{50}	(95% CI)	*N*	CTR/AMY_1R	CTR/AMY_3R
Pramlintide	9.78	0.10	2	9.08	0.36	3	6.84	0.07	4	871	173
sCT	9.47	0.04	2	9.06	0.13	3	9.29	0.05	4	1	1
NN1213	8.51	0.12	2	7.95	0.32	3	6.23	0.58	4	190	52

Binding data reported as $pIC_{50} \pm$ 95% CI. Values are presented from 2–4 experiments on human (h), canine (c), and rat (r) AMY_1R, AMY_3R, or CTR transiently expressed in BHK cells. Ratio represents amylin receptor (AMYR) selectivity and is determined by dividing the IC_{50} values for the CTR with that of the relevant AMYR. NN1213 is the designated nomenclature for peptide **21**. pIC_{50}, negative logarithm of the half maximal inhibitory concentration of the peptide.

Table S7. Species-Specific Potencies on CTR, AMY_1R, and AMY_3R

	$hAMY_1R$		$hAMY_3R$		hCTR		Ratio	
Compound	pEC_{50}	(95% CI)	pEC_{50}	(95% CI)	pEC_{50}	(95% CI)	CTR/AMY_1R	CTR/AMY_3R
Pramlintide	10.12	0.26	10.62	0.18	9.43	0.28	5	16
sCT	10.77	0.28	11.01	0.17	10.71	0.24	1	2
NN1213	9.57	0.59	9.78	0.64	8.07	0.54	32	51
	$cAMY_1R$		$cAMY_3R$		cCTR		Ratio	
	pEC_{50}	(95% CI)	pEC_{50}	(95% CI)	pEC_{50}	(95% CI)	CTR/AMY_1R	CTR/AMY_3R
Pramlintide	10.77	0.31	10.88	0.23	10.90	0.32	1	1
sCT	10.61	0.34	11.16	0.24	11.27	0.40	0.2	0.8
NN1213	9.87	0.45	9.74	0.69	9.39	0.38	3	2
	$rAMY_1R$		$rAMY_3R$		rCTR		Ratio	
	pEC_{50}	(95% CI)	pEC_{50}	(95% CI)	pEC_{50}	(95% CI)	CTR/AMY_1R	CTR/AMY_3R
Pramlintide	11.34	0.36	11.47	0.15	9.96	0.12	24	32
sCT	11.42	0.25	11.81	0.16	12.01	0.15	0.3	0.6
NN1213	10.69	0.57	10.32	0.64	8.17	0.89	336	144
	$mAMY_1R$		$mAMY_3R$		mCTR		Ratio	
	pEC_{50}	(95% CI)	pEC_{50}	(95% CI)	pEC_{50}	(95% CI)	CTR/AMY_1R	CTR/AMY_3R
Pramlintide	ND	-	9.4	0.34	8.51	0.23	-	8
sCT	ND	-	11.0	0.13	11.11	0.12	-	1
NN1213	ND	-	9.3	0.29	7.89	0.17	-	24

$mAMY_1R$, mouse AMY_1R; ND, not done.

There was a very good correlation between the binding affinities of the tested analogs between the three species. sCT bound with high affinity on both amylin receptors and CTRs, whereas pramlintide and NN1213/peptide **21** were AMY_1R and AMY_3R selective in all three species (Table S6).

sCT activated both AMYRs and CTRs with high potency, whereas pramlintide and NN1213/peptide **21** were AMY_1R and AMY_3R selective in all four species (Table S7). The mouse was only tested for the AMY_3R and the CTR.

Overall, there was a good correlation between the binding data and the functional data. However, for all analogs, the functional potencies (pEC_{50} values) appear to be stronger than the apparent binding affinities (pIC_{50} values) obtained from the competitive binding assay. This difference is most likely because the transfected cells express very high levels of receptors (thus have spare receptors). The degree of spare receptors influences the compound potencies and binding affinities differently in each assay.[3] Specifically, if there is a high degree of spare receptors, the compounds will appear more potent. On the other hand, the degree of spare receptors has little influence on the binding affinities. Thus, it is expected that the binding displacement (pIC_{50}) values will be higher than the functional potencies in this experimental setting.

For the canine receptors, the degree of selectivity in the functional assays for the AMYRs is very low for NN1213/peptide **21**, and pramlintide is nonselective. The switch in potency for NN1213/peptide **21** is only 2-fold for the $cAMY_1R$ and 3-fold for the $cAMY_3R$ when compared to the cCTR. In the binding assay for the canine receptors, the switch in apparent binding affinity is 73- and 40-fold, respectively, for the $cAMY_1R$ and $cAMY_3R$, when compared to the cCTR.

The most likely scenario for this observation is that the cCTRs are expressed at very high levels compared with rCTR and hCTR. Alternately, canine RAMP (cRAMP)1 and cRAMP3 may be expressed poorly. If this is the case, the ratio between cRAMP and cCTR will be very low and the system will then express more cCTRs than canine AMYRs, even when both RAMP and CTR are co-transfected. Accordingly, the apparent "enigma" that pramlintide and peptide **21** appear amylin receptor selective in the canine binding assay but not in the functional canine assay, is most likely explained by the fact that, in the binding experiment, we can measure specifically for the AMYRs and the CTRs, whereas in the functional assay, we measure a pool of AMYRs and CTRs when we assess the potencies in the "amylin receptors". In the binding assay, it is possible to measure specifically for both the AMYRs and CTRs since the radiolabeled ^{125}I-rat-amylin tracer binds very specifically to the AMYRs and not the CTRs. Likewise, the radiolabeled human calcitonin tracer only binds the CTRs. Thus, the results in the amylin binding assay will not be influenced by the excess presence of CTRs. However, in the functional assay, there will always be a pool of CTRs in the cells, even when we co-express RAMP and CTRs to general AMYRs. If the RAMP:CTR ratio is low, as we expect it is in the canine model, it will not be possible to detect ligand selectivity because very few will be AMYRs and thus the contribution from CTRs will contaminate the results.

Thus, the functional data are harder to translate directly since the functional potencies can be affected by receptor expression level, the RAMP/CTR ratio, the receptor's ability to couple to the G_s protein, and the analog binding affinity.

Functional potencies obtained in a cAMP assay are reported as $pEC_{50} \pm 95\%$ CI. Values are presented from five experiments on human, canine, and rat Amylin 1, Amylin 3, and CTRs transiently expressed in BHK cells. The mouse Amylin 3 and CTRs were analyzed by expressing the receptors using the BacMam system to transduce the receptor constructs into HeLa cells. The assay conditions are described in the Method section. In both assays, the "ratio" represents the AMYR selectivity and is determined by dividing the EC_{50} values for the hCTR with that of $hAMY_3R$.

S5: *In vivo* Methods.

Pharmacokinetic Evaluation. *Rats:* Male Sprague-Dawley rats (Taconic, Europe; body weight approximately 400 g) were group-housed under controlled temperature conditions (20 °C ± 2 °C). Blood was collected from the sublingual vein and rats were acclimated to handling on three separate occasions prior to blood collection. Iv bolus injections were performed via a 23 G Venflon catheter in the tail vein of five rats. Sc injections were performed in the neck of another group of five rats. The dosing volume was 2 mL/kg. Blood samples were obtained pre-dose as well as at 5 min, 15 min, 30 min, 1 h, 2 h, 4 h, 6 h, 12 h, 24 h, 30 h, 48 h, 72 h, and 96 h after dosing. All blood samples were collected into test tubes containing EDTA for stabilization and kept on ice until centrifugation. Each blood sample was 100 µL. Plasma was separated from whole blood by centrifugation and was stored at –20 °C or lower until analysis.

Rabbits: Female rabbits (New Zealand white, Charles River; 21–23 weeks; 2.5–3.5 kg) were group-housed (10 per cage) in normal day–night rhythm from arrival and had free access to food and water. The rabbits were treated against coccidiosis during their 5-week acclimation period upon arrival. Iv dosing was performed as a bolus injection into an ear vein with a dosing volume of 0.1 mL/kg. The dosing solution was at room temperature before injection. Venous blood sampling was performed directly through puncture of an ear vein using a needle. A full plasma concentration–time profile was obtained from each animal. Blood samples were taken pre-dose and at 5 min, 30 min, 1 h, 1.5 h, 3 h, 6 h, 8 h, 24 h, 48 h, 96 h, 168 h, and 216 h post-dose. Blood (300 µL) was collected directly into test tubes containing EDTA buffer (Microvette CB 300 K2E). Samples were kept on wet ice until centrifugation (10 min, 4 °C, 3000 rpm). Plasma (100 µL) was transferred immediately to Micronic tubes and kept at –20 °C until analysis.

Minipigs: Male Göttingen minipigs (Ellegaard, Denmark; approximately 8–9 months old; 22.1–24.6 kg) were housed in groups until the experiment. Two permanent central venous catheters were implemented in *vena cava caudalis* in each animal 1 week prior to the study. The pigs were then housed in individual pens. Minipigs were fasted 24 h pre-dosing with free access to water. Iv bolus injections were given through one of the catheters. Post iv administration, the catheter was flushed with 10 mL of sterile saline. The dosing volume was 0.05 mL/kg and the dosing solution was at room temperature before injection. Blood samples were preferably taken from the catheter not used for iv bolus injection. After each blood sample, the catheter was flushed with 5 mL of sterile 0.9% NaCl. A full plasma concentration–actual time profile was obtained from each animal with blood samples taken pre-dose and at 5 min, 15 min, 30 min, 45 min, 1 h, 1.5 h, 2 h, 3 h, 4 h, 6 h, 8 h, 10 h, 24 h, 48 h, 72 h, 96 h, 120 h, 168 h, 192 h, 216 h, 240 h, 264 h, 288 h, 336 h, 360 h, 384 h, 418 h, 442 h, and 456 h post-dose. All blood samples (0.8 mL each) were collected into test tubes containing EDTA for stabilization and kept on ice until centrifugation. Plasma was separated from whole blood by centrifugation and stored at –20 °C or lower until analysis.

Dogs: Male beagle dogs (HsdRcc: DOBE from Harlan Gannat, France; approximately 6 months old; 9.6–12 kg) were group-housed in a pen of approximately 8.5 m^2 with sawdust as bedding material. The temperature in the room was set at 20–23 °C and the relative humidity to 30–70%. The room was illuminated to give a cycle of 12 h of light and 12 h of slight light for orientation. Two venflons were inserted in each cephalic vein, one for dosing and one for blood sampling. After dosing, the venflon was removed and only the venflon for blood sampling was left and kept open with heparin saline (10 IU heparin pr. mL isotonic saline) for blood sampling for the initial profile (15 min, 30 min, 45 min, 1 h, 1.5 h, 2 h, 2.5 h, 3 h, 3.5 h, 4.5 h, 6 h, 8 h, and 10 h post-dose). Blood samples taken at 24, 48, 72, 96, 120, 144, 168, 192, 216, 240, 264, 288, 312, and 336 h post-dose were drawn from the jugular vein. During blood sampling, dogs were placed on a table and fixated by an animal technician. Approximately 800 mL of blood was collected in 1.5 mL EDTA eppendorf tubes or EDTA vacutainers for plasma. Blood samples were kept on wet ice for a maximum of 20 min before centrifugation at 4 °C, 4 min, 1300 x g. Plasma was immediately transferred to micronic tubes and stored at –20 °C until analysis.

Plasma analysis: Plasma samples were analyzed by a commercially available kit (Millipore, Human Amylin ELISA, cat. #EZHA-52-K). Briefly, the assay was a two-sited ELISA using two antibodies against human amylin. A monoclonal antibody was immobilized to the surface of microplate wells. Plasma samples, dosing solution or calibrators (NN1213) were applied to the appropriate wells and

incubated for 1 h. The wells were then emptied and washed, and an alkaline phosphatase-conjugated antibody was added and incubated for an additional 2 h. After another washing step, a substrate solution (4-methylumbelliferyl phosphate) was added and incubated for 15 min. Finally, a stop solution was applied and the plate was read on a fluorescent reader with an excitation/emission wavelength of 335 nM/460 nM. The level of signal was proportional to the concentration of NN1213 in the plasma samples. The lower limit of quantification (LLOQ) was 100–500 pM.

Pharmacokinetics analysis: Plasma concentration–time profiles were analyzed by non-compartmental analysis using Phoenix WinNonlin Professional 6.3 (Pharsight, Mountain View, CA, US). Calculations were performed using individual concentration–actual time values from the animals. The area under the curve (AUC) was calculated and given as AUCinf_pred, unless otherwise stated. The method used to calculate AUC was Linear Log Trapezoidal Interpolation. The percentage of extrapolated AUC was less than 25% in all individuals. The sc bioavailability was calculated as (AUC/Dose)sc/(AUC/Dose)iv; iv dose: 2.5 nmol/kg, sc dose: 5 nmol/kg. The given mean values are all arithmetic except for $T_{½}$ and T_{max}, which are given as harmonic mean and median, respectively. Pre-dose values below LLOQ were set to 0. Post-dose values below LLOQ were treated as missing and excluded from the non-compartmental analysis.

Efficacy Evaluation. Conversion details for the dosing of peptide **21** for *in vivo* studies are given in Table S8.

Table S8. Conversion Table for Doses of Peptide **21** (**MW** = 4553.2 g/mol) Used for *In vivo* Studies

µg/kg → nmol/kg		nmol/kg → µg/kg	
µg/kg	nmol/kg	nmol/kg	µg/kg
0.1	0.022	0.1	0.455
0.3	0.066	0.3	1.37
1	0.220	1	4.6
3	0.66	3	13.7
10	2.2	10	46
30	6.6	30	137
100	22	100	455

Acute rat food intake assay: The acute effects of amylin analogs on appetite in rats were measured using the Feedwin system (Ellegaard Systems, Faaborg, Denmark), in which up to 32 rats (male Sprague Dawley, Taconic Europe; 200–250 g) were single-housed for individual registration of food consumption. Diet: Altromin 1324 (Brogaarden, Denmark). The rats were housed using a reverse light cycle (12 h of light and 12 h of dark) and acclimatized to the reverse light cycle and single-housing for at least 5 days prior to testing under controlled temperature conditions (20 °C ± 1 °C). The rats were dosed immediately before the lights were turned off at 10 am. Food intake was recorded for 48 h after dosing. Hereafter, the rats were euthanized by inhalation of CO_2. Test substances were formulated in 2 mM acetate, 250 mM glycerol, 0.025% tween-20, pH 4. Accumulated food intake was calculated for the three periods 0–24, 24–48, and 0–48 h, respectively. In figure 4, mean accumulated food intake (0-48 hours) in treatment groups was compared to vehicle using one-way analysis of variance using an alpha level of 5%. Significance is denoted by asterisks.

Acute dog food intake assay: Appetite was assessed in beagle dogs (45 weeks old) after a single sc administration of 3, 10, 30, 100, and 300 nmol/kg. The study was conducted as an ascending dose study with the same dogs being dosed at different dose levels with a minimum of 14 days between dose events. The dogs were housed in groups but were trained to eat separately for two meals per day (diet: VetXX, Specific CCD, Dechra, UK). Appetite was assessed by weighing the excess food that was not eaten within 30 min. Appetite was assessed for 4 days after dosing. In figure 4, mean accumulated food intake from dosing and 48 hours hereafter in treatment groups were compared to vehicle using one-way analysis of variance using an alpha level of 5%. Significance is marked by asterisks (or ns: not significant).

DIO rat weight loss assay: The sub-chronic effects from NN1213 on food intake and body weight in rats were assessed in DIO rats. Obesity was induced by feeding the rats (male Sprague Dawley, Vital River, Beijing) a high fat diet (Research Diets [HF D12451, 45% fat]) for at least 28 weeks prior to testing. The rats were housed individually under a controlled light cycle (light from 6 pm–6 am) under controlled temperature conditions (22 °C ± 1 °C). The DIO rats were allocated to groups based on body weight and fat mass, and were dosed once daily with a sc administration of vehicle or 0.7, 2.2, 6.6, and 22 nmol/kg of NN1213 (3, 10, 30, and 100 µg/kg, $n = 8$/group). A control group of low-fat-diet fed (Research Diets [HF D12450, 10% fat]), age-matched rats ($n = 8$) were also dosed with vehicle. Food consumption and body weight were recorded daily. Body composition was assessed by magnetic resonance scan (EchoMRI) and was measured 6 days prior to the first dose and at day 21. Body fat and lean mass were calculated as change from baseline.

S6: *In vivo* Results.

Pharmacokinetic Evaluation.

Table S9. Pharmacokinetics of NN1213 in Animal Models

Species	*n*	Route of administration	Dose (nmol/kg)	$T_{1/2}$ (h)
Rat	5	Iv	2.5	24
Rabbit	9	Iv	2	49
Minipig	4	Iv	4.8	116
Dog	3	Iv	6	76

Table S10. Body Weight and Food Intake Comparisons in the DIO Rat Study

	Absolute body weight (g)		Relative body weight (% of initial body weight)	Cumulative food intake (% of vehicle HFD)
Treatment	Day 0	Day 21	Day 21	Day 21
Vehicle, HFD	747.1 ± 32.5	758.3 ± 36.8	101.3 ± 0.6	100.0 ± 6.2
NN1213, 3 µg/kg (0.7 nmol/kg)	737.5 ± 36.8	705.5 ± 38.4	95.3 ± 0.7*	74.1 ± 4.1*
NN1213, 10 µg/kg (2.2 nmol/kg)	728.8 ± 19.4	664.4 ± 19.3	91.2 ± 0.7*	57.1 ± 3.6*
NN1213, 30 µg/kg (6.6 nmol/kg)	748.6 ± 31.4	682.4 ± 27.7	91.3 ± 0.4*	61.8 ± 1.6*
NN1213, 100 µg/kg (22 nmol/kg)	737.4 ± 13.7	667.8 ± 11.9	90.7 ± 0.9*	59.2 ± 4.0*
Vehicle, low fat diet	667.5 ± 12.9	669.6 ± 15.0	100.1 ± 0.5	86.1 ± 2.4

Data are mean ± SEM; *$p < 0.05$, repeated measures two-way analysis of variance, Dunnett's *post hoc* versus vehicle, HFD; HFD, high fat diet.

S7: Materials and Methods of Peptide Synthesis.

All chemicals were of analytical grade or higher.

Triisopropylsilane, N,N´diisopropylcarbodiimide, and formic acid (FA; ≥98%) were from Sigma-Aldrich, Chemie GmBH (Steinheim, Germany). Acetonitrile (LiChrosolve), trifluoroacetyl (TFA), and diethyl ether were purchased from Merck KGaA (Darmstadt, Germany). Water came from a MilliQ equipment (Advantage A10) from Millipore (Molsheim, France). Standard Fmoc-aminoacids, resins and coupling reagents, and OxymaPure were from Novabiochem (Darmstadt, Germany) or Protein Technologies (Tucson, US). N-methylpyrrolidone, dimethyl formamide, and piperidine were from Biosolve (Dieuze, France). Fmoc-Rink amide polystyrene resin, amino acids were all purchased from Merck Millipore (Novabiochem, Strasbourg France). Acetonitrile with 0.1% TFA (buffer B; alternatively, 10–30% buffer B) over 40 min on a SymmetryPrep C18 19 × 300 mm, 7 µm column (Waters Corporation, Milford, US) eluting at 20 mL/min. For some peptides, the purification protocol was shortened to a gradient from 10–30% buffer B, 15–35% buffer B, or 20–40% buffer B in 20 min. Analysis of purity of the peptides was performed using Waters Acquity ultra-performance liquid chromatography (UPLC) system, with Waters Acquity TUV detector 214 nM and 254 nM. Column details and gradients used are in Table S9. For some peptides, the molecular weights were determined using matrix-assisted laser desorption and ionization TOF MS, recorded on a Microflex or Autoflex (Bruker-Daltonics, Bremen, Germany). A matrix of α-cyano-4-hydroxy cinnamic acid was used. Alternatively, characterization was performed by UPLC–MS on a setup consisting of a Waters Acquity

UPLC system connected to an LCT Premier XE mass spectrometer from Micromass, or by HPLC–MS on an Agilent 1200 series HPLC connected to an Agilent 6230 TOF system using solvent A 0.1% FA in H_2O and solvent B 0.1% FA in acetonitrile. All the purified analogs were dissolved in 80% DMSO with 20% H_2O as stock solutions. All peptide stocks for *in vitro* assays were quantified to a final concentration close to 200 µm by using a chemiluminescent nitrogen detector (Thermo Scientific Vanquish) or charged aerosol detector (Thermo Scientific Ultimate 3000) before doing an *in vitro* assay. The measured concentration was used in data analysis.

Table S11. Purity (%), Retention Time, and Found Mass (Average) of Peptides

Peptide	HPLC purity (%)	System	Retention time (min)	Calculated MW	ESMS found mass $[M+H]^{3+}$	ESMS found mass $[M+H]^{4+}$	MALDI found mass
1	100	B4	8.71	4397.00	1465.9	1099.7	
2	100	B4	8.67	4396.02	1465.5	1099.4	
3	95.5	UPLC02	13.27	4554.17	1518.7	1139.3	
4	95.1	UPLC02	13.48	4554.17	1519.7	1139.3	
5	100	B4	8.67	4512.09	1504.7	1128.8	
6	100	B4	8.58	4512.09	1504.7	1128.7	
7	97.5	B4	8.80	4512.09	1505.4	1129.0	
8	98.2	UPLC02	13.36	4512.09	1504.4	1128.7	
9	100	B4	8.82	4569.19	1524.1	1143.0	
10	97.8	B5	4.27	4671.23	1558.8	1168.7	
11	98.0	B5	4.22	4378.94	1460.4	1095.6	
12	96.7	UPLC02	12.18	4405.96	1469.6	1102.5	
13	98.8	B5	4.30	4394.98	1465.9	1099.7	
14	95.1	B5	4.40	4394.98	1465.9	1099.7	
15	92.9	B5	4.59	4568.15			4568.9
16	95.2	B5	4.04	4450.02	1484.2	1113.7	
17	97.0	B5	4.90	4430.98			4429.1
18	99.3	B4	8.67	4549.11			4546.3
19	96.7	B5	4.34	4489.01	1497.3	1123.3	
20	100	B4	8.66	4438.01	1480.4	1110.3	
21	100	B4	8.59	4553.09	1518.5	1139.6	

ESMS, electrospray mass spectrometry; $[M+H]^{+}$, analyte and hydrogen protonated adduct.

LC-MS Method. *LC-system:* Waters ACQUITY UPLC H-Class BEH C18 Column, 1.7 µm, 2.1 mm x 50 mm; 5–95% 0.1% FA over 6 min; detector, Waters Xevo G2-XS QTof; detector setup, ionization method, electrospray; scanning range, 50–4000 amu; operating mode, MS resolution mode, positive mode; voltage, capillary, 3.00 kV; sample cone, 40 V; source, 80 V; temperature, source, 150 °C and desolvation, 500 °C; scan time: 0.50 s; interscan delay: 0.014 s.

MALDI (mass spectrometry method): For some peptides, the MWs were determined using MALDI and ionization TOF MS, recorded on a Microflex or Autoflex (Bruker Daltonics, Bremen, Germany). A matrix of α-cyano-4-hydroxy cinnamic acid was used.

UPLC Method. *UPLC02. LC system:* Waters ACQUITY UPLC BEH Shield RP18 Column, 150 mm; UPLC Eluent A, 0.05% TFA in Milli-Q water; UPLC Eluent B, 0.05% TFA in acetonitrile; UPLC Gradient B, 5–95% (20 min runtime); column temperature, 50 °C; wavelength, 214 nM; flow rate, 0.4 mL/min.

B4. LC system: Waters ACQUITY UPLC Symmetry300 C18 Column, 5 µm, 3.9 mm x 150 mm; UPLC Eluent A, 0.05% TFA in MQ water; UPLC Eluent B, 1% TFA in acetonitrile; UPLC Gradient B, 5–95% (15 min runtime); column temperature, 42 °C; wavelength, 214 nM; flow rate, 1.0 mL/min.

B5. LC system: Waters ACQUITY UPLC BEH Shield RP18 Column, 1.7 µm, 2.1 x 150 mm column; UPLC Eluent A, 70% acetonitrile/30% water; UPLC Eluent B, 0.2 M Na_2SO_4, 0.04 M H_3PO_4, 10% acetonitrile; UPLC Gradient, 40% A in 1 min; 40 → 70% A (7 min runtime); column temperature: 42 °C; wavelength: 214 nM; flow rate: 0.35 mL/min.

Channel Description ACQUITY TUV ChA 214nm

Date Acquired 1/27/2011 4:02:41 PM CET

Vial : 1:2 Vol. : 5.00 ul Acq Method Set : 09_B4_1_214nm

Date Processed 1/27/2011 4:22:45 PM CET

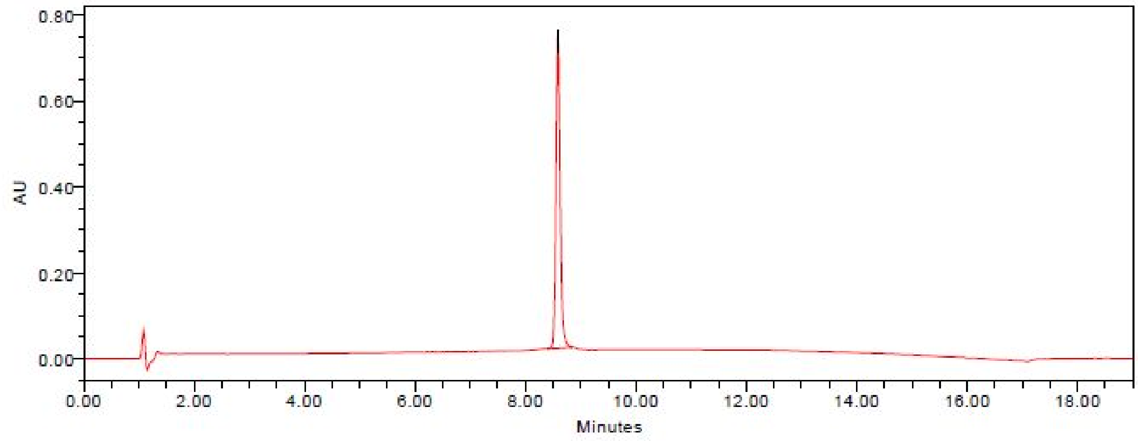

	RT	Area	Height (μV)	% Area
1	8.589	3874923	722538	100.00

A: 0.05% TFA in water
B: 0.05% TFA in acetonitrile
Gradient : 5 ----> 95% B, 16min, 0.4ml/min
Acquity UPLC BEH130, 1.7um, 2.1 x 150 mm column
column own temp. = 40 °C

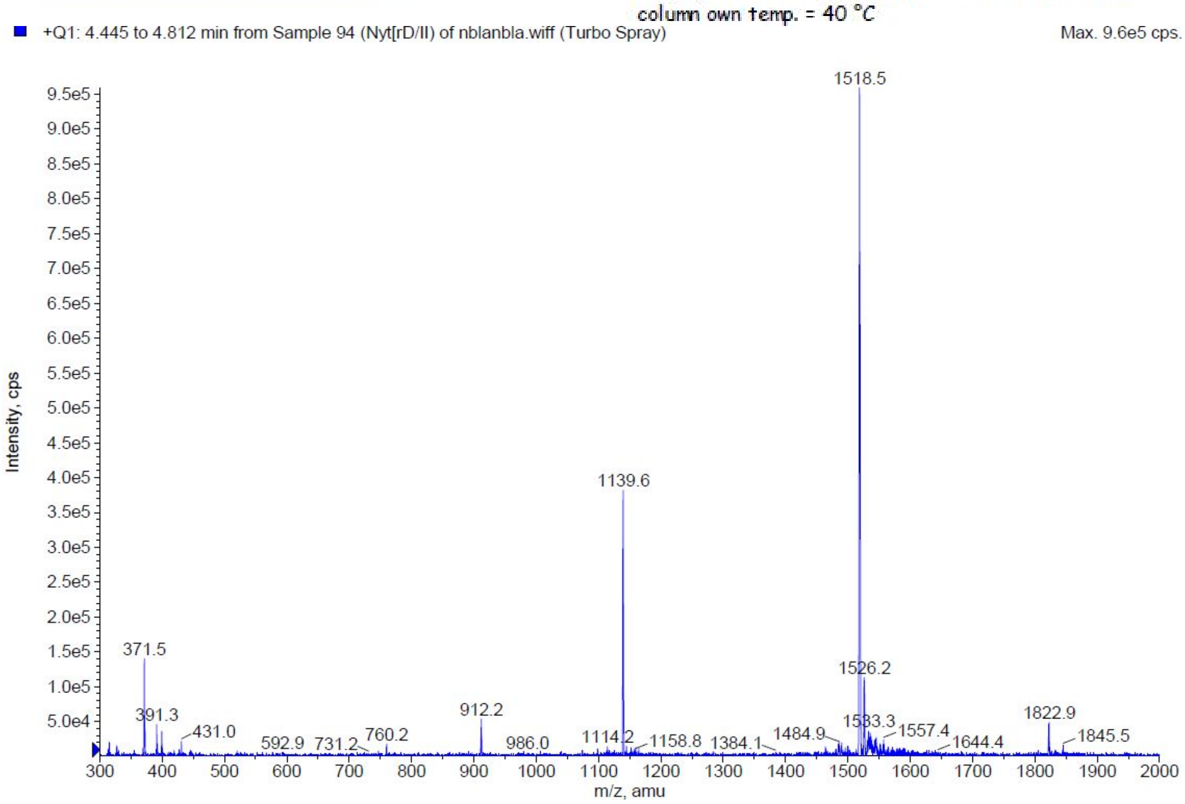

Figure S1. Representative UPLC Chromatogram (A) and LC-MS (B) for Peptide **21** (NN1213). AU, absorbance unit; RT, retention time.

Solubility versus pH Profiles. Solubility profiles were assessed by mixing 50 μL aliquots of a 500 μM aqueous solution of each compound with 50 μL of 100 mM pH-adjusted buffer solutions (lactate pH 3−5; bis-tris-propane pH 6−8) to a nominal concentration of 250 μM. Samples were left overnight at room temperature to reach solubility equilibrium (as determination of concentration by HPLC did not change after this timepoint) and subsequently centrifuged to isolate the supernatant. The peptide concentration in the supernatant was determined by UPLC using an ACQUITY UPLC column (bridged ethylsiloxane/silica hybrid C181.7 μm−2.1×50 mm) with a flow rate of 0.45 mL/min at 40°C and detection at 214 nm. A gradient combining eluent A (0.05% TFA in water) and eluent B (0.05% TFA in acetonitrile) was applied (%A/%B: 0 min: 95/5; 0−½ min: linear to 90/10; ½−2½ min: linear to 35/65; 2½−3 min: linear 0/100; 3−4 min: 0/100; 4−4½ min: linear to 95/5; and5 min: 95/5). Measured values at or above 200 μM were reported as ">200 μM", whereas measured values below 200 μM were reported as determined.

S8: SAR Screening Assay Methods.

Functional Assays (Human and Rat). Brief descriptions of the methods for functional assays used to determine potencies for SAR analysis are described below. Curves and EC_{50} values were calculated in GraphPad Prism using nonlinear regression with pEC_{50} values expressed as –log EC_{50}. Data are expressed as pEC_{50} values, with 95% CIs. EC_{50} values were used to calculate the potency ratios in order to express the selectivity of compounds for the AMYR (complexed with RAMP3; AMY_3R]) versus the CTR.

Human Calcitonin Receptor Luciferase Assay. A BHK 570 cell line stably transfected with the hCTR, and a cAMP-responsive element luciferase reporter gene (Hollex-1 cell line, obtained from Zymogenetics described in US patent 5622839) were used in this functional assay. In this cell line, the hCTR activity is reflected in the luciferase intensity occurring in response to amylin. The cells were cultured in growth medium (DMEM with 10% FBS, 1% penicillin/streptomycin, and 1 mM Na-pyruvate). Methotrexate (250 nM) and neomycin (500 μg/mL) were used as selection markers for luciferase and CTR, respectively. Cells at approximately 80–90% confluence were washed with PBS and lifted from the plates with Versene. After centrifugation (2 min, 1300 rpm), the cell pellet was dissolved in 10% DMSO, 30% FBS, and 60% growth medium (see above), and frozen (–80 °C) until utilization. The day before the experiment, cells were thawed, washed, and seeded in 100 μL growth medium (as described above) on a white 96-well culture plate (20000 cells/well). After incubation overnight at 37 °C and 5% CO_2, the growth medium was replaced with 50 μL/well of assay medium (DMEM [without phenol red]), Glutamax, 10% FBS, and 10 mM HEPES, pH 7.4). Further, 50 μL/well of sample diluted in assay buffer was added. After 3 h of incubation at 37 °C and 5% CO_2, the medium was removed and replaced by 100 μL/well PBS and 100 μL/well SteadyLite (Perkin Elmer 6016759). The plates were sealed and incubated at room temperature for 30 min. Finally, luminescence was measured on a TopCounter (Packard) in single photon counting mode.

Human Amylin-3 Receptor Luciferase Assay. To generate a stable clone expressing the AMY_3R, the Hollex-1 cells were further transfected with RAMP3 and pcDNA3.1/hygromycin; the latter was used as a selection marker. Briefly, the transfection was carried out in a T75 flask seeded with 1,250,000 cells the day before transfection. The cells were transfected with 9 μg RAMP3 cDNA, 1 μg pcDNA3.1/hygromycin, and 25 μL FuGENE 6 (Promega E2692). Hereafter, a stable clone expressing RAMP3 was selected. The stable clone was cultured in DMEM with 10% FBS, 1%

penicillin/streptomycin, and 1 mM Na-pyruvate. Methotrexate (250 nM), neomycin (500 µg/mL), and hygromycin (400 µg/mL) were used as selection markers for luciferase, CTR, and RAMP3, respectively. Cells at approximately 80–90% confluence were washed with PBS and lifted from the plates with Versene. After centrifugation (2 min, 1300 rpm), the cell pellet was dissolved in 10% DMSO, 30% FBS, and 60% growth medium (see above), and frozen (–80 °C) until utilization. The day before the experiment, cells were thawed, washed, and seeded in 100 µL growth medium (as described above) on a white 96-well culture plate (20000 cells/well). After incubation overnight at 37 °C and 5% CO_2, the growth medium was replaced by 50 µL/well assay medium (DMEM [without phenol red]), Glutamax, 10% FBS, and 10 mM HEPES, pH 7.4). Further, 50 µL/well of sample diluted in assay buffer was added. After 3 h of incubation at 37 °C and 5% CO_2, the medium was removed and replaced by 100 µL/well PBS and 100 µL/well SteadyLite (Perkin Elmer 6016759). The plates were sealed and incubated at room temperature for 30 min. Finally, luminescence was measured on a TopCounter (Packard) in single photon counting mode.

Rat CTR cAMP Assay. BHK tk-ts 13 cells (a temperature-sensitive clone of the BHK cell line described in Croce et al.[4]) were transiently transfected with rCTR using FuGENE 6 (Promega E2692) in a ratio of 1 µg cDNA:1.5 µL FuGENE 6, according to the manufacturer's recommendations. Cells were grown in DMEM with 10% FBS and 1% penicillin/streptomycin. Approximately 24 h after transfection, the cells were harvested and frozen (–80 °C) until utilization. On the day of experimentation, cells were thawed, washed twice, and then re-suspended in PBS buffer (2% HSA, 0.5% Tween-20). Cells were seeded (100,000 cells/well) into 96-well FlashPlates (Perkin Elmer) with samples or standard. Here, 50 µL cell of the suspension was added to the FlashPlates containing 50 µL of test-compound or reference compound (2% HSA, 0.5% Tween 20). The mixture was shaken for 5 min and allowed to stand for 25 min at room temperature. The reaction was stopped with 100 µL detection mix pro well (detection mix; 11 mL detection buffer and 100 µL [~2 µCi] cAMP [^{125}I] Tracer). The plates were then sealed with plastic, shaken for 30 min, and allowed to stand overnight (or for at least 2 h), and scintillation was measured in a TopCounter (2 min/well). In general, the assay procedure described in the FlashPlate kit-protocol was followed (FlashPlate cAMP assay [NEN™ Life Science Products cat no SMP004]). The cAMP amount was determined using a standard curve.

Rat AMY$_3$R cAMP Assay. BHK tk-ts 13 cells were transiently transfected with rCTR (150 µg rCTR cDNA pr 10,000,000 cells) and rat RAMP3 (the cDNA ratio was 1 µg CtR cDNA pr 1.5 µg rat RAMP3) using 2.5 µL FuGENE 6 per µg cDNA. Cells were grown in DMEM with 10% FBS and 1% penicillin/streptomycin. Approximately 24 h after transfection, the cells were harvested and frozen (–80 °C) until utilization. On the day of experimentation, cells were thawed, washed twice, and then re-suspended in PBS buffer (2% HSA, 0.5% Tween 20). Cells were seeded (100,000 cells/well) into 96 well FlashPlates (Perkin Elmer) with samples or standard. Here, 50 µL cell of the suspension was added to the FlashPlates containing 50 µL of test-compound or reference compound (2% HSA, 0.5% Tween-20). The mixture was shaken for 5 min and allowed to stand for 25 min at room temperature. The reaction was stopped with 100 µL detection mix pro well (detection mix; 11 mL detection buffer and 100 µL [~2 µCi] cAMP [^{125}I] Tracer). The plates were then sealed with plastic, shaken for 30 min, and allowed to stand overnight (or for at least 2 h), and scintillation was measured in a TopCounter (2 min/well). In general, the assay procedure described in the FlashPlate kit-protocol was followed (Plate cAMP assay [NEN™ Life Science Products cat no SMP004]). The cAMP amount was determined using a standard curve.

Binding Assays (Human and Rat). Brief descriptions of the methods used to determine binding affinities for SAR analysis are described below. Curves and IC_{50} values were plotted and calculated in GraphPad Prism using nonlinear regression (one site binding competition analysis), with pIC_{50} values expressed as –log IC_{50}. Data (Table 4 and Table 11) are expressed as pIC_{50} values, with 95% CIs. IC_{50} values were used to calculate the binding affinity ratios in order to express the selectivity of compounds for the AMY_3R versus the CTR.

CTR Binding Assay. The binding assay was performed using scintillation proximity assay (SPA) beads (RPNQ0001) from PerkinElmer and cell membranes containing either the hCTR or rCTR. BHK tk-ts 13 cells were transiently transfected with the hCTR or rCTR and cultured as described above. Membranes were prepared in the following way: the cells were rinsed with PBS and incubated with Versene for approximately 5 min before harvesting. The cells were flushed with PBS and the cell-suspension was centrifuged for 5 min at 1000 rpm. Cells were homogenized (Ultra Turrax) in a buffer containing 20 mM Na-HEPES and 10 mM EDTA (pH 7.4) and centrifuged at 20000 rpm for 15 min. The resulting pellet was re-suspended, homogenized, and centrifuged (20000 rpm, 15 min) in a buffer containing 20 mM Na-HEPES and 0.1 mM EDTA (pH 7.4, buffer 2). The resulting pellet was re-suspended in buffer 2 and protein concentration was measured (BCA protein assay, Pierce). The homogenate was kept cold during the whole procedure. The membranes were stored at –80 °C until use.

The binding assay was performed in a 384-well Optiplate (PerkinElmer) in a total volume of 40 μL. Membranes were mixed with SPA beads in a 1:1 ratio. The final concentration of SPA beads was 0.05 mg/well. Test compounds were dissolved in DMSO and further diluted in assay buffer (50 mM HEPES, pH 7.4, 1 mM $CaCl_2$, 5 mM $MgCl_2$, 0.1% ovalbumin, and 0.02% Tween-20). Radioligand ^{125}I-Calcitonin (NEX422 PerkinElmer) was dissolved in assay buffer and added to the Optiplate at a final concentration of 75 pM/well (30000 cpm/10 μL). The final mixture was incubated for 120 min at 25 °C prior to centrifugation (1500 rpm, 10 min). Samples were analyzed on TopCounter (Packard).

AMY_3R Binding Assay. The binding assay was performed using SPA beads (RPNQ0001) from PerkinElmer and cell membranes containing either the $hAMY_3R$ or $rAMY_3R$. BHK tk-ts 13 cells were transiently transfected with the hCTR or rCTR and hRAMP3 or rRAMP3 at an equimolar ratio (1:3 μg) and cultured as described above. Membranes were prepared in the following way: the cells were rinsed with PBS and incubated with Versene for approximately 5 min before harvesting. The cells were flushed with PBS and the cell-suspension was centrifuged for 5 min at 1000 rpm. Cells were homogenized (Ultra Turrax) in a buffer containing 20 mM Na-HEPES and 10 mM EDTA (pH 7.4), and centrifuged at 20000 rpm for 15 min. The resulting pellet was re-suspended, homogenized, and centrifuged (20000 rpm, 15 min) in a buffer containing 20 mM Na-HEPES and 0.1 mM EDTA (pH 7.4, buffer 2). The resulting pellet was re-suspended in buffer 2 and protein concentration was measured (BCA protein assay, Pierce). The homogenate was kept cold during the whole procedure. The membranes were stored at –80 °C until use.

The binding assay was performed in a 384-well Optiplate (PerkinElmer) in a total volume of 40 μL. Membranes were mixed with SPA beads in a 1:1 ratio. The final concentration of SPA beads was 0.05 mg/well. Test compounds were dissolved in DMSO and further diluted in assay buffer (50 mM HEPES, pH 7.4, 1 mM $CaCl_2$, 5 mM $MgCl_2$, 0.1% ovalbumin, and 0.02% Tween-20). Radioligand ^{125}I-rAMY (NEX448 PerkinElmer) was dissolved in assay buffer and added to the Optiplate at a final

concentration of 50 pM/well (20000 cpm/10 μL). The final mixture was incubated for 120 min at 25 °C prior to centrifugation (1500 rpm, 10 min). Samples were analyzed on a TopCounter (Packard).

S9: SAR Screening Assay Results.

Table S12. *In vitro* hCTR and $hAMY_3R$ Functional Results for Analogs 1–9

	Functional assays															
	Luciferase (human receptor assay)								cAMP (rat receptor assay)							
	$hAMY_3R$				hCTR				$rAMY_3R$				rCTR			
Analog	EC_{50} (pM)	pEC_{50}	95% CI	*n*	EC_{50} (pM)	pEC_{50}	95% CI	*n*	EC_{50} (pM)	pEC_{50}	95% CI	*n*	EC_{50} (pM)	pEC_{50}	95% CI	*n*
Pramlintide	5	11.31	0.03	343	70	10.15	0.05	264	4	11.40	0.19	10	1,822	8.74	0.19	8
sCT	1.6	11.79	0.14	20	2.9	11.54	0.19	19	0.52	12.28	0.34	3	0.14	12.85	0.21	2
1	180	9.74	0.13	2	697	9.16	0.06	2	463	9.33	ND	1	14,340	7.84	ND	1
2	1,535	8.81	0.43	2	1,371	8.86	0.18	2								
3	319	9.50	0.14	4	3,005	8.52	0.1	4								
4	267	9.57	0.03	2	672	9.17	0	2								
5	219	9.66	0.11	4	2,579	8.59	0.15	4								
6	161	9.79	0.16	8	2,477	8.61	0.19	8	643	9.19	ND	1	860,600	6.07	ND	1
7	200	9.70	0.18	2	1,380	8.86	0.01	2								
8	390	9.41	0.17	2	2,190	8.66	0.12	2								
9	1,061	8.97	0.01	2	8,309	8.08	0.02	2								

Data are pEC_{50} ± 95% CIs on AMY_3R and CTR.

Table S13. *In vitro* hCTR and hAMY$_3$R Binding Results for Analogs 3, 5–7

	Binding assays															
	Human receptors								Rat receptors							
	hAMY$_3$R				hCTR				rAMY$_3$R				rCTR			
Analog	IC$_{50}$ (pM)	pIC$_{50}$	95% CI	*n*	IC$_{50}$ (pM)	pIC$_{50}$	95% CI	*n*	IC$_{50}$ (pM)	pIC$_{50}$	95% CI	*n*	IC$_{50}$ (pM)	pIC$_{50}$	95% CI	*n*
Pramlintide	114	9.94	0.09	52	1,492	8.83	0.1	51	122	9.91	0.11	45	5,834	8.23	0.12	44
sCT	89	10.05	0.2	11	66	10.18	0.12	13	28	10.56	0.37	10	30	10.53	0.16	10
3	5,095	8.29	ND	1	74,108	7.13	0.28	3	4,690	8.33	ND	1	355,750	6.45	0.21	2
5	1,121	8.95	0.27	2	30,233	7.52	0.24	3	1,300	8.89	ND	1	105,572	6.98	0.62	2
6	355	9.45	0.2	6	19,476	7.71	0.15	8	217	9.66	0.376	4	96,915	7.01	0.2	5
7	1,125	8.95	0.08	2	11,662	7.93	0.2	2	1,002	9.00	ND	1	7,545	8.12	1.22	2

Data are pIC$_{50}$ ± 95% CIs on AMY$_3$R and CTR.

Table S14. *In vitro* Potency for Human and Rat AMY$_3$R and CTR; Analogs 10–21

	Functional assays															
	Luciferase (human receptor assay)								cAMP (rat receptor assay)							
	hAMY$_3$R				hCTR				rAMY$_3$R				rCTR			
Analog	EC$_{50}$ (pM)	pEC$_{50}$	95% CI	*n*	EC$_{50}$ (pM)	pEC$_{50}$	95% CI	*n*	EC$_{50}$ (pM)	pEC$_{50}$	95% CI	*n*	EC$_{50}$ (pM)	pEC$_{50}$	95% CI	*n*
10	432	9.36	0.03	2	10,000	8.00	0.00	2	1,788	8.75	ND	1	141,900	6.85	ND	1
11	186	9.73	0.28	3	1,993	8.70	0.10	3	1,288	8.89	ND	1	16,210	7.79	ND	1
12	47	10.33	0.20	2	314	9.50	0.17	2								
13	310	9.51	0.08	2	2,609	8.58	0.04	2	599	9.22	ND	1	30,370	7.52	ND	1
14	364	9.44	ND	1	5,459	8.26	ND	1	754	9.12	ND	1	37,160	7.43	ND	1
15	352	9.45	ND	1	18,260	7.74	ND	1								
16	217	9.66	ND	1	2,522	8.60	ND	1								
17	151	9.82	0.18	4	2,492	8.60	0.17	4	926	9.03	ND	1	16,810	7.77	ND	1
18	350	9.46	0.11	2	6,500	8.19	0.01	2								
19	222	9.65	ND	1	1,322	8.88	ND	1								
20	340	9.47	0.21	3	5,204	8.28	0.03	3								
21	177	9.75	0.17	17	5,210	8.28	0.22	15	262	9.58	ND	1	102,500	6.99	ND	1

Data are pEC$_{50}$ ± 95% CIs on AMY$_3$R and CTR.

Table S15. *In vitro* Binding Affinity for Human and Rat AMY_3R and CTR; Analogs 10–21

	Binding assays pIC_{50}															
	$hAMY_3R$				hCTR				$rAMY_3R$				rCTR			
Analog	IC_{50} (pM)	pIC_{50}	95% CI	*n*	IC_{50} (pM)	pIC_{50}	95% CI	*n*	IC_{50} (pM)	pIC_{50}	95% CI	*n*	IC_{50} (pM)	pIC_{50}	95% CI	*n*
10	1,138	8.94	ND	1	102,400	6.99	ND	1	3,006	8.52	ND	1	349,700	6.46	ND	1
11	589	9.23	0.13	3	11,927	7.92	0.15	3	1,171	8.93	0.14	3	51,410	7.29	0.19	3
12	100	10.00	ND	1	1,088	8.96	ND	1	176	9.75	ND	1	5,976	8.22	ND	1
13	1,139	8.94	ND	1	73,390	7.13	ND	1	1,715	8.77	ND	1	89,170	7.05	ND	1
14	885	9.05	0.22	3	42,342	7.37	0.33	2	920	9.04	0.12	3	55,644	7.25	0.28	2
15	213	9.67	0.27	3	21,148	7.67	0.20	3	372	9.43	0.13	3	32,737	7.48	0.27	3
16	662	9.18	0.06	2	19,280	7.71	ND	1	1,768	8.75	0.02	2	96,440	7.02	ND	1
17	484	9.32	0.15	7	14,097	7.85	0.21	6	1,293	8.89	0.16	7	49,815	7.30	0.30	6
18	2,746	8.56	0.22	3	52,262	7.28	0.20	3	1,240	8.91	0.26	3	71,016	7.15	0.24	3
19	344	9.46	ND	1	15,460	7.81	ND	1	203	9.69	ND	1	17,110	7.77	ND	1
20	745	9.13	0.22	3	22,905	7.64	0.40	3	449	9.35	0.27	3	50,048	7.30	0.32	3
21	561	9.25	0.12	13	42,049	7.38	0.17	12	290	9.54	0.17	12	134,993	6.87	0.16	12

Data are $pIC_{50} \pm$ 95% CIs on AMY_3R and CTR.

S10: ThT Fluorescence Time Courses

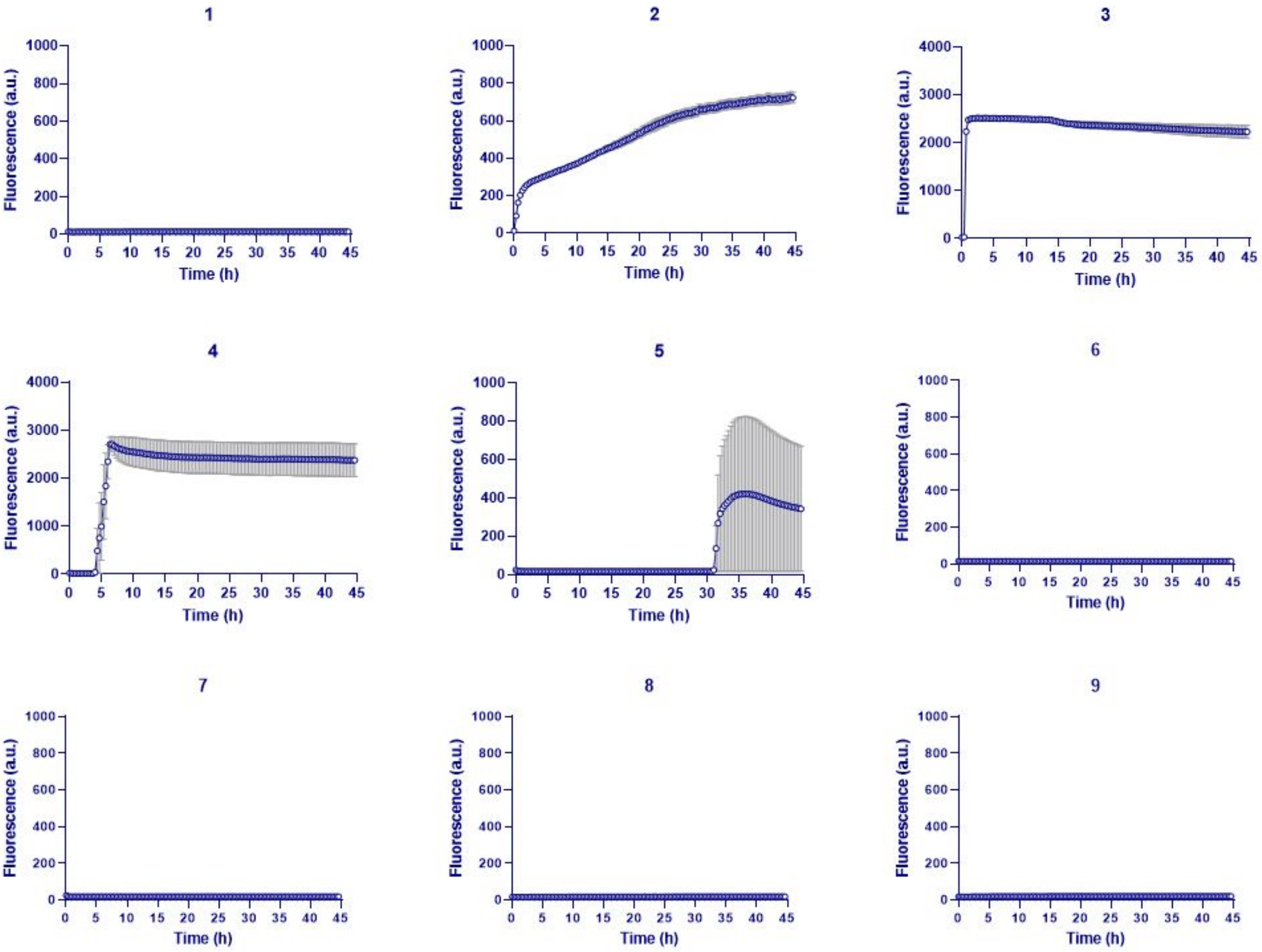

Figure S2. ThT Fluorescence Time Courses of Peptides 1–9 at pH 7.5 (see also Table 6). Each time course represents the average of n wells ± SEM, n=4.

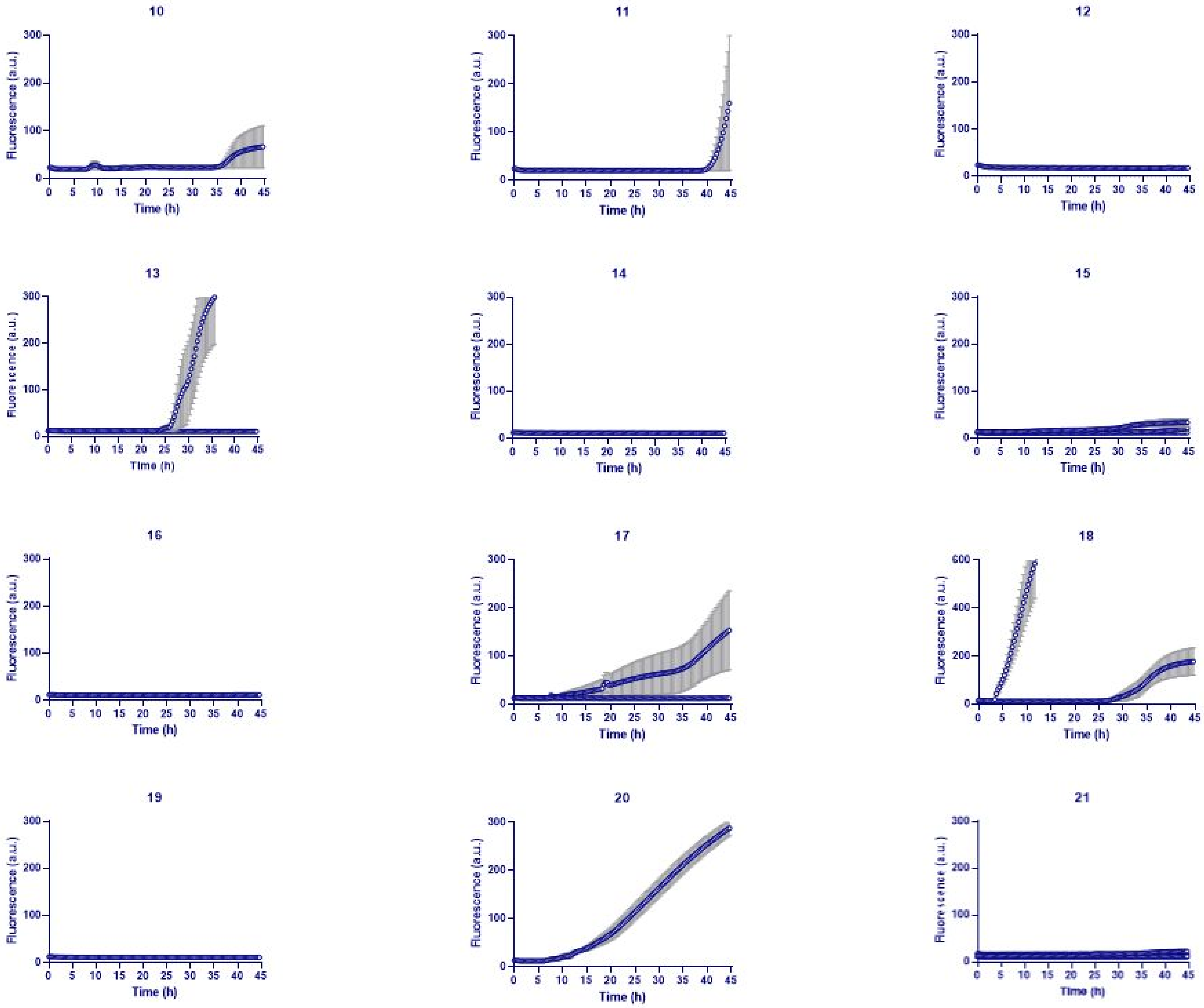

Figure S3. ThT Fluorescence Time Courses of Peptides 10–21 at pH 4.0 (see also Table 9). Some peptides have been tested more than once; hence, more than one time course is presented. Each time course represents the average of n wells ± SEM, n≥4.

REFERENCES

(1) Motulsky, H. J.; Brown, R. E. Detecting outliers when fitting data with nonlinear regression - a new method based on robust nonlinear regression and the false discovery rate. *BMC Bioinformatics* **2006**, *7*, 123. DOI: 10.1186/1471-2105-7-123

(2) Kruse, T.; Hansen, J. L.; Dahl, K.; Schäffer, L.; Sensfuss, U.; Poulsen, C.; Schlein, M.; Hansen, A. M. K.; Jeppesen, C. B.; Dornonville de la Cour, C.; Clausen, T. R.; Johansson, E.; Fulle, S.; Skyggebjerg, R. B.; Raun, K. Development of cagrilintide, a long-acting amylin analogue. *J. Med. Chem.* **2021**, *64* (15), 11183–11194. DOI: 10.1021/acs.jmedchem.1c00565

(3) Zhu, B. T. The competitive and noncompetitive antagonism of receptor-mediated drug actions in the presence of spare receptors. *J. Pharmacol. Toxicol. Methods* **1993**, *29* (2), 85–91. DOI: 10.1016/1056-8719(93)90055-j

(4) Croce, C. M.; Talavera, A.; Basilico, C.; Miller, O. J. Suppression of production of mouse 28S ribosomal RNA in mouse-human hybrids segregating mouse chromosomes. *Proc. Natl. Acad. Sci. U. S. A.* **1977**, *74* (2), 694–697. DOI: 10.1073/pnas.74.2.694
